# Body image phenomenology in reports of psychedelic and meditative experiences: main themes and associations with body attitudes

**DOI:** 10.1093/nc/niag062

**Published:** 2026-09-21

**Authors:** Hilla Väyrynen, Nanna Strid, Emma Ylönen, Jarno Tuominen, Pilleriin Sikka, Jussi Jylkkä

**Affiliations:** Independent researcher, Oulu, Finland; Department of Psychology and Speech-Language Pathology, University of Turku, Assistentinkatu 7, 20500 Turku, Finland; Department of Psychology and Speech-Language Pathology, University of Turku, Assistentinkatu 7, 20500 Turku, Finland; Department of Cognitive Neuroscience and Philosophy, University of Skövde, Högskolevägen 3, 549 55 Skövde, Sweden; Department of Psychology and Speech-Language Pathology, University of Turku, Assistentinkatu 7, 20500 Turku, Finland; Department of Psychology and Speech-Language Pathology, University of Turku, Assistentinkatu 7, 20500 Turku, Finland; Department of Psychology and Speech-Language Pathology, University of Turku, Assistentinkatu 7, 20500 Turku, Finland; Department of Cognitive Neuroscience and Philosophy, University of Skövde, Högskolevägen 3, 549 55 Skövde, Sweden; Department of Anesthesiology, Perioperative & Pain Medicine, Stanford University School of Medicine, 300 Pasteur Drive, Room H3580, MC 5640, Stanford, CA 94305, United States; Department of Psychology, Stanford University, 450 Jane Stanford Way, Building 420, Stanford, CA 94305, United States; Department of Psychology, Åbo Akademi University, Arken, Tehtaankatu 2, 20500 Turku, Finland; Department of Philosophy, University of Turku, Assistentinkatu 7, 20500 Turku, Finland

**Keywords:** body image, embodiment, meditation, psychedelics, phenomenology

## Abstract

Psychedelics and meditation can facilitate transformative experiences with acute changes in sensory processing and self-perception, but research about their relation to narrative and affective-attitudinal body image remains limited. We examined body image themes in psychedelic and meditative experience narratives, compared these between experience types, and examined their associations with body attitudes. We qualitatively analyzed retrospective reports of participants’ most personally meaningful psychedelic and meditation experiences (psychedelics: *N* = 90; meditation: *N* = 48). Using regression models, we examined associations between identified themes, current body appreciation, and perceived changes in body dissatisfaction, body appreciation, and body connection, and whether individuals with eating disorder traits reported distinctive themes or body attitude changes. Body image-related narratives most commonly involved reduced sense of bodily self and heightened sensory sensitivity. Psychedelic reports more frequently described disembodiment, the loss of body as an anchor, while meditation reports more often involved boundary dissolution while maintaining embodied presence. 3,4-Methylenedioxymethamphetamine (MDMA) reports described heightened embodiment more often than classical psychedelic reports. Both heightened embodiment and disembodiment narratives were associated with perceived improvements in body dissatisfaction and body connection. Positive affective-attitudinal body image themes were associated with perceived improvements in body connection. Participants with current eating disorder traits, including histories of eating disorders, more frequently reported positive affective-attitudinal body image themes and greater perceived reductions in body dissatisfaction. These findings suggest that psychedelic and meditative body image phenomenology, particularly embodiment-related, can be perceived as meaningful for subjective body attitudes.

## Introduction

Psychedelics and meditation can facilitate deeply transformative experiences with phenomenological similarities, such as a flexible and reduced sense of self, disembodiment, unity, and personally meaningful insights (Barrett et al. 2018, Millière et al. 2018, Letheby 2022, Holas and Kamińska 2023, Timmermann et al. 2023, Zanesco et al. 2023, Simonsson et al. 2024, Jylkkä et al. 2025). Psychedelic-assisted and mindfulness-based interventions show promise in improving body attitudes in those with body image disorders, with individual participants in psychedelic trials noting that the subjective experience played a role (Butryn et al. 2013, Breeksema et al. 2020, Brewerton et al. 2022, Gopan et al. 2024, Peck et al. 2024). Yet, how the body image phenomenology in psychedelic or meditative experience narratives relates to perceived changes in body attitudes has not been studied previously. This is an important perspective to take, because subjective experience is at the core of body image (Raoul and Grosbras 2023).

Body image means the body as an object of consciousness (Gallagher 2005). It is a multifaceted construct, most commonly divided into perceptual and affective-attitudinal domains. Perceptual body image represents beliefs and impressions about one’s physical form, and affective-attitudinal body image, the emotionally charged meanings and evaluations through which the body is viewed, referred to as body attitudes (Cash and Smolak 2011).

Central to body image is embodiment, which encompasses the direct awareness of and connection to one’s body, relating to it through a first-person perspective, and attuning to its signals (Riva 2018, Piran et al. 2020, Burychka et al. 2021). Higher embodiment is linked to flexible and adaptive body attitudes, whereas deficits in this area are linked to self-objectification, body dissatisfaction, and shame (Ainley and Tsakiris 2013, Burychka et al. 2021, Naraindas and Cooney 2023, Naraindas et al. 2025).

Embodiment requires the ability to integrate immediate multisensory bottom-up information, such as interoceptive and proprioceptive cues, and bind these into a unitary bodily self-experience (Tsakiris et al. 2007, Blanke 2012). In body image disturbance, attunement to body-related bottom-up signals is often compromised, creating a chasm between the lived body and body image (Riva and Dakanalis 2018, Brizzi et al. 2023). Thus, an adaptive balance between sensory bottom-up and sense-making top-down processes is not achieved, and rigid inner models, such as beliefs about appearance or self-worth, dominate body attitudes and perceptions.

In eating disorders (ED), inflexible body attitudes, self-beliefs, and emotional coping strategies form a key transdiagnostic dynamic (Abdoli et al. 2025). Current pharmacological treatments tend to target diagnosis-specific symptoms by modulating food-related impulses and attenuating anxiety-related signalling. World Federation of Societies of Biological Psychiatry guidelines list olanzapine for weight gain in anorexia nervosa, fluoxetine for compulsivity in bulimia nervosa, and lisdexamfetamine or topiramate for impulsivity in binge-eating disorder (Himmerich et al. 2023). While these treatments can reduce behavioural symptoms, core features such as body and self-image disturbance often persist and relapse rates remain substantial (Keel and Brown 2010, Eshkevari et al. 2014, Glashouwer et al. 2019).

In contrast, classical and non-classical psychedelics as well as meditation increase openness to multisensory and emotional bottom-up stimuli and reduce top-down dominance (Carhart-Harris and Friston 2019, Laukkonen and Slagter 2021, Avram et al. 2024, Dai et al. 2025). Such conditions are proposed to alter bodily self-experience, potentially opening a window of plasticity to revise inflexible body image-related beliefs (Riva 2018, Ho et al. 2020, Ledwos et al. 2023). Quantitative altered states of consciousness (ASC) literature has recognized altered embodiment as a recurring core dimension in psychedelic states since the 1960s, describing depersonalization, changes in self-world boundaries, and changed percepts of bodily proportions due to altered interoceptive processing (Studerus et al. 2010, Fort et al. 2025). However, central aspects of body image, such as its narrative and affective-attitudinal domains, remain largely unstudied.

Psychedelics are a heterogeneous family of substances. Classical psychedelics (e.g. lysergic acid diethylamide [LSD], psilocybin, and dimethyltryptamine [DMT]), are defined by serotonin 2A receptor (5-HT2A) agonism and monoamine modulation, whereas non-classical psychedelics act through diverse mechanisms, e.g*. N*-methyl-D-aspartate (NMDA) antagonism in dissociatives such as ketamine, monoamine release and modulation in empathogens such as MDMA, and multi-target action (including kappa-opioid agonism and NMDA antagonism) in atypical compounds such as ibogaine. Rather than through pharmacology, the rationale for grouping these together is based on phenomenology involving features like attentional expansion, ego dissolution, and hyperassociativity (O'Donnell et al. 2023), though the breadth of this experience-based definition remains contested (Nichols et al. 2023). Thus, the definition of “psychedelic” is somewhat context-dependent. Because we examined the meaningfulness of subjective body image phenomena rather than drug-specific mechanisms, we followed the experience-based definition of psychedelics, while acknowledging that body image alterations likely differ in character across these substance classes.

The transformative potential of psychedelic experiences may be amplified by increased neuroplasticity. Classical psychedelics, ketamine, and MDMA increase brain-derived neurotrophic factor (BDNF) signalling and are proposed to open a phase of plasticity resembling childhood critical learning periods (Moliner et al. 2023, Aboharb et al. 2025, Agnorelli et al. 2025). Interestingly, increased peripheral BDNF has also been reported in meditators (Gomutbutra et al. 2020).

Recent longitudinal and clinical studies have found classical psychedelics and meditation to facilitate improvements in affective-attitudinal body image in clinical and naturalistic settings (de Wet et al. 2020, Barba et al. 2024, Gopan et al. 2024, Peck et al. 2024). Classical psychedelics and meditation have been linked to acutely enhanced interoception and positively felt body connection (Swift et al. 2017, Bornemann et al. 2021, de Lima-Araujo et al. 2022, Barba et al. 2024, Mograbi et al. 2024). Large doses of classical psychedelics or ketamine as well as deep meditative states are associated with disembodiment, such as out-of-body experiences, feelings of floating, or not having a body (Berkovich-Ohana et al. 2013, Hirschfeld et al. 2023, Sarasso et al. 2024, Vizeli et al. 2024). Changes in perceptual body image, like seeing one’s appearance in the mirror as changed or experiencing the body or its parts changing in size, are mainly reported with classical psychedelics, particularly in body dysmorphic disorder (Hanes 1996, Girn and Christoff 2018).

In previous psychedelic studies, individual participants have reported perceiving body-related experiences as important for their body image by allowing them to revise the subjective meaning of their body (Swift et al. 2017, Bornemann et al. 2021). For example, in a psilocybin study, an anorexia nervosa patient described how disembodiment prompted them to grieve how much time they had spent hyperfixating on a part “that just holds my being” (Peck et al. 2024). In a qualitative study about ayahuasca experiences among individuals in ED recovery, a participant reported coming to see her body as a “gift” that should be honored, and another participant described a vision of herself transitioning from a “rotting, decaying skeleton” to a “beautiful full-bodied woman,” which encouraged her to start gaining weight (Lafrance et al. 2017). These narratives imply that body image-related phenomenology in psychedelic experiences can be perceived as profoundly meaningful.

In meditation, altered bodily experiences, such as feelings of floating and body boundaries vanishing, may be more frequent than commonly recognized (Galante et al. 2024, Wright et al. 2024). While meditation literature has not discussed the subjective importance of altered bodily self-experiences, such phenomena could be similarly meaningful regardless of how they are facilitated (Yaden et al. 2017, Millière et al. 2018). Moreover, psychedelics and meditation are two of the widespread routes to voluntarily induce non-ordinary experiences in somewhat overlapping populations (Simonsson and Goldberg 2023). Examining these narratives side-by-side allows for a richer understanding of the meaningfulness of altered body image phenomenology for body attitudes.

The dataset in this study has been analyzed in two previous studies, a qualitative study about reports of psychedelic and meditative insights (Jylkkä et al. 2025), and a natural language processing analysis comparing the full reports (Kallio-Mannila et al. 2025). Responses to the questions about body image have not been analyzed previously.

The aim of this study was to identify body image-related themes in narrative reports of transformative psychedelic and meditative experiences, compare these between facilitation methods and drug types, and to examine the identified themes’ associations with perceived changes in three body attitudes: body dissatisfaction, body appreciation, and body connection. Although body connection is a feature of embodiment, it can also be seen as a body attitude, because it describes a comfortable and non-objectifying relationship with one’s body. We measured current body appreciation to examine whether reporting specific themes was associated with current positive affective-attitudinal body image. Secondly, we examined whether individuals with ED traits, including histories of ED, reported specific themes more than those with no such traits, and whether they perceived changes in body attitudes distinctively. While this was a correlative study, such affective-attitudinal body image problems are crucially important for context.

## Method

The research questions, data gathering approach, and analysis were preregistered (https://osf.io/5pa8f). Preregistration and transparency are detailed in Analysis. The preregistered research questions were as follows:

1. What are key body image-related themes in narrative reports of psychedelic and meditative peak experiences?
2. Do the theme frequencies differ between psychedelic and meditative narratives, or between different psychoactive substances or meditation styles?
3. Are body image-related themes associated with current body appreciation or perceived body attitude changes following the experience?
4. Are ED traits associated with different body image-related themes in the reports or different perceived body attitude changes?

The study was approved by the Research Ethics Committee at Åbo Akademi University (#15092022) and followed the GDPR regulations regarding privacy and data management, as well as the declaration of Helsinki regarding ethical principles of research.

### Participants

The data were collected internationally with two separate anonymous online surveys for psychedelic and meditation reports between October 2022 and March 2023. The participants were recruited through social media, psychedelic and meditation focused online communities, and by contacting relevant associations directly. Participants had to be at least 18 years old. All participants gave their informed consent in written form. The inclusion of both psychedelic and meditation groups was disclosed to the participants after completing the survey. No compensation was given for participation.

### Materials

#### Open report prompt

Participants were asked to recall their “most personally meaningful or important” psychedelic or meditation experience, referred to as the Experience. The task has been used in studies to retrospectively probe transformative experiences (Kangaslampi et al. 2020, Strickland et al. 2024). Narrative reports were collected with an open-ended prompt “Please write down everything you experienced during the Experience (what happened, where, who was present, thoughts, feelings, images, scenarios) as accurately and in as much detail as possible. Remember that every detail is important”. The prompt was applied from dream research and aimed to collect reports in as much detail as possible (Sikka 2019). The open report prompt was administered before the more detailed questions to avoid bias.

#### Thematic questions

After the open report, participants were asked, “Did you perceive your body in a different way or have any personally meaningful thoughts, feelings or insight about it?” with three categorical reply options: “Yes,” “No,” and “Don’t know,” followed by the prompt: “If yes, please describe these aspects in English below”. Additionally, thematic questions of insights and experiences of connectedness or alienation were included, analyzed elsewhere (Jylkkä et al. 2025).

#### Eating disorder traits

By measuring ED traits, we wanted to track individuals with body image-related rigidity or concern and identify those with lifetime histories of EDs. ED traits were measured with the Eating Disorder Screen for Primary Care (ESP; Cotton et al. 2003) and used as a continuous variable in regression analyses to capture variation. ESP is a short self-report measure for effective patient screening with four questions, answered dichotomously with Yes or No: (i) “Are you satisfied with your eating patterns?,” (ii) “Do you ever eat in secret?,” (iii) “Does your weight affect the way you feel about yourself?,” and (iv) “Do you suffer, or have you ever suffered in the past with an eating disorder?” The score is the sum of the items with a range of 0–4. A cut-off of 2 or more is considered a basis for further investigation, with 100% sensitivity and 71% specificity (Cotton et al. 2003). In this sample, ESP demonstrated questionable internal consistency with Cronbach’s Alpha, α = 0.59, likely due to the small number of items and their dichotomous response format.

#### Current body appreciation

Current body appreciation was measured with Body Appreciation Scale-2 (BAS-2; Tylka and Wood-Barcalow 2015). BAS-2 is a unidimensional 10-item self-report scale measuring positive affective-attitudinal body image features, such as holistic acceptance and respect for one’s body. The items consist of 10 statements (e.g*.* “I respect my body,” “I feel good about my body,” and “I am attentive to my body’s needs”) which are answered on a 5-point Likert scale ranging from 1 (Never) to 5 (Always). The final score is the mean of the 10 items. In this sample, BAS-2 demonstrated excellent internal consistency with Cronbach’s α = 0.93.

#### Perceived body attitude changes

The participants retrospectively assessed subjective changes in their body attitudes following the Experience, referred to as perceived body attitude changes. These were collected with the question, “As a result of the Experience, have you noticed any persisting increases or decreases in the following?” Answers were given on a 5-point Likert scale from 1 (‘Decreased a lot’) through 3 (‘Not changed (stayed the same)’) to 5 (‘Increased a lot’). Three body attitudes were assessed: (a) “Your critical thoughts and feelings about your body?,” signifying body dissatisfaction; (b) “Your acceptance and appreciation for your body?,” signifying body appreciation; and (c) “Your sense of being ‘at home’ in your body?,” signifying body connection (Cash and Smolak 2011, Tylka and Wood-Barcalow 2015, Piran et al. 2020). These were examined as individual variables.

### Procedure

The first part of the survey collected demographic data: sex (female/male/other), age, education and income levels, religion, country of origin, psychiatric diagnoses, psychedelic use history, and meditation habits. Here, ESP was used to assess participant’s ED traits.

The second part collected information about the background of the Experience. This was the only part of the survey that differed between the psychedelic and meditation surveys. In the psychedelic survey, participants gave details about the substance(s) used to facilitate the Experience, listed as: “LSD or analogues,” “Psilocybin or ‘magic mushrooms’,” “Ayahuasca,” “N,N-DMT,” “5-MeO-DMT or Bufo Alvarius,” “Mescaline or psychoactive cacti,” “Ibogaine,” “*Salvia divinorum,*” “MDMA, ‘ecstasy’ or ‘molly’,” “Ketamine,” and “Cannabis.” In the meditation survey, participants gave details about meditation techniques, listed as: “mindfulness,” “silent sitting or lying down,” “voicework, e.g. chanting,” “bodywork, e.g. yoga,” and/or “breathwork (intentional manipulation of breath)”. Selecting several listed drugs or meditation techniques was permitted and additional details could be given in a text box. Participants determined how much time had passed since the Experience. Details about set and setting, dose, intention, and preparedness were collected but not analyzed in this study.

The third part of the surveys consisted of writing the open report of the Experience. After completion, participants were presented with the thematic question and wrote a thematic report about bodily aspects. Thematic reports about insight experiences and connection/alienation were collected but not used in this study. All thematic questions were presented in a randomized order. Next, participants assessed perceived changes in body attitudes and other psychological well-being factors. The final part consisted of the BAS-2 and other psychometric scales. Completing the survey took ~30–60 min. The full surveys are available in the Open Science Framework (https://osf.io/whma3/).

### Analysis

#### Qualitative analyses

Qualitative analysis was preregistered as a combination of predefined and data-driven themes. Three body image domains were preregistered as main themes: (1) perceptual body image, (2) affective-attitudinal body image, and (3) embodiment. Their subthemes and other themes were data-driven, developed to reflect the narrative content more accurately.

We used a coder reliability approach to thematic analysis (TA) to qualitatively analyze the reports (Boyatzis 1998, Braun and Clarke 2021). Based on reading the reports iteratively against body image literature and existing psychometric scales, H.V. compiled the Codebook for Body-Related Experiences with instructions, theme definitions, and hypothetical examples. The codebook is presented in Supplementary Material S1: https://osf.io/whma3/.

In accordance with the coding reliability approach to TA, we only coded explicit body-related statements and did not interpret beyond these (Boyatzis 1998). Cases with absent reports and multiple experiences were excluded. Reports of participants replying “No” to the thematic question about the presence of bodily aspects were removed from qualitative analysis but later included in statistical analysis as a theme representing the lack of bodily reference.

Two independent coders, N.S. and E.Y., coded the reports. They were provided with the codebook, all reports in a randomized order, and an empty data matrix for inserting ratings. The coders read each report and marked in the data matrix whether each theme was present in the report (0/1), or whether the case met exclusion criteria. When a theme was present, the sequence exhibiting said theme was quoted. Multiple themes could co-occur within a single report. We studied the measures of agreement on the presence of coded themes with Cohen’s Kappa, and settled discrepant codings in a consensus coding meeting between H.V., N.S., and E.Y.

#### Statistical analyses

Given the partially data-driven design, the preregistration did not name individual themes yet specified their statistical analysis. This consisted of dummy-coding all identified themes, presenting their frequencies descriptively, comparing these between experience types (psychedelics/meditation; drug and meditation types), and using these as variables in regression models to examine their associations with perceived body attitude changes, current body appreciation, and ED traits, adjusting for age and sex.

Based on the characteristics of the data, we made several adjustments to ensure meaningful comparisons. Sex was studied as a binary variable due to the small number of participants identifying as “other” (*n* = 4). These participants were removed from regression models. When comparing theme frequencies by drug type, three cannabis reports were omitted from “non-classical psychedelics,” as the remaining reports in this category were about MDMA only, and aggregating these was not considered meaningful in this context. Moreover, we conducted separate regression models for psychedelic and meditation reports when examining associations between the themes and ED traits, because three themes were exclusive to one experience type (psychedelics/meditation), resulting in perfect separation when this was used as an independent variable.

Deviating from the preregistration, we did not compare theme frequencies between meditation styles due to limited variability in the data, as most meditation respondents reported silent meditation or mindfulness. We studied ED traits as a continuous variable (ESP score) instead of group comparisons to improve statistical power and capture variance.

We conducted statistical analyses with RStudio 2025. We studied the internal consistency of standardized questionnaires in these data with Cronbach’s Alpha. To examine demographic data, we used Student’s *t*, Pearson’s *χ*^2^ and Mann–Whitney *U* tests. We compared the occurrences of themes between the meditation and psychedelic reports with Pearson’s *χ*^2^ tests and their effect sizes with Cramer’s *V*. For comparing the occurrences of the themes between drug types in the psychedelic reports, we used Fisher’s exact tests. When a main theme had subthemes, only these were used in the regression models.

We studied the associations between ED traits and the themes in the reports with 19 binary logistic regression models using each dummy-coded theme as the dependent variable in either psychedelic reports (10 models) or meditation reports (9 models) and the ESP score as the independent variable. We examined goodness-of-fit with Hosmer-Lemeshow tests.

We used three ordinal logistic regression models to study the associations between the themes in the reports and perceived body attitude changes (body dissatisfaction, body appreciation, and body connection), and to examine whether participants with ED traits perceived body attitude changes distinctively. We used each body attitude change variable as the dependent variable in their respective models, and all 11 dummy-coded themes, experience type (psychedelic/meditation), and ESP score as independent variables.

We performed a linear regression model to examine associations between the themes in the reports and current body appreciation. BAS-2 score was used as the dependent variable and all 11 dummy-coded themes, experience type (psychedelic/meditation), and ESP score as independent variables. Because these analyses were preregistered, we did not apply mechanical multiple comparison corrections such as false discovery rate, and instead, considered it suitable to interpret the results based on context, theoretical coherence, and previous findings (Hooper 2025).

## Results

### Demographics

In total, 213 participants responded, 147 to the psychedelic survey, and 66 to the meditation survey. Of these, 19 cases were missing data due to a survey platform malfunction, 8 did not provide reports, and 7 reported several experiences. These participants were removed from analyses. Thus, 179 participants were included, 117 via the psychedelic survey and 62 via the meditation survey.

Qualitative analysis included reports from 90 (77%) psychedelic and 48 (77%) meditation respondents who categorically stated that their experience was or may have been body-related. The participants who categorically stated that their experience was not body-related, 27 (23%) psychedelic and 14 (23%) meditation respondents, were added to quantitative analysis. The presence of body-related experiences is presented in Table 1. The responses were almost identical between psychedelic and meditation respondents (*χ*^2^ = 0.02, *P* = .99).

**Table 1:** Presence of body-related experiences.

Body-related experience (*χ* ^2^ = 0.02, *P* = .99)	Psychedelics (*n* = 117*)*		Meditation (*n* = 62)	
	*n*	*%*	*n*	*%*
Yes	78	66.7	42	67.7
No	27	23.1	14	22.6
Don’t know	12	10.3	6	9.7

The presence of body-related experiences was assessed with the thematic question “Did you perceive your body in a different way or have personally meaningful thoughts, feelings or insights about your body?,” answered categorically.

Psychedelic respondents were younger than meditation respondents (*M* = 39.5, *SD* = 13.4 vs. *M* = 51.1, *SD* = 14.4, *t* = 5.39, *P* < .001). Demographic data of the participants are presented in Table 2. Participants were from all continents and educational backgrounds. The meditation survey had more American respondents; however, the majority of all participants were European and had university degrees. More meditators practiced Buddhism, and more psychedelic respondents identified as atheist, but the most common denomination among both was “spiritual but not religious”.

**Table 2:** Demographics of the participants*.*

Demographic	Psychedelics (*n* = 117)		Meditation (*n* = 62)	
Gender (*χ* ^2^ = 5.27, *P* = .07)	*n*	*%*	*n*	*%*
Female	43	36.8	29	46.8
Male	73	62.4	30	48.4
Other	1	0.9	3	4.8
**Education level** (*U* = 3189.0, *P* = .17)	*n*	*%*	*n*	*%*
Primary education	1	0.9	0	0.0
Lower secondary education	4	3.4	1	1.6
Higher secondary education	12	10.3	2	3.2
Vocational education	6	5.1	4	6.5
University: Bachelor’s degree	34	29.1	18	29.0
University: Master’s degree	40	34.2	24	38.7
University: Doctoral degree	14	12.0	9	14.5
Other	6	5.1	4	6.5
**Income level** (*U* = 3050.0, *P* = .07)	*n*	*%*	*n*	*%*
Much below average	9	7.7	10	16.1
Below average	21	17.9	15	24.2
Average	24	20.5	11	17.7
Above average	53	45.3	21	33.9
Much above average	10	8.5	5	8.1
**Religion** (*χ*^2^ = 45.0, ***P* < .001**)	*n*	*%*	*n*	*%*
Agnostic	20	17.1	4	6.5
Atheist	24	20.5	2	3.2
Buddhist	2	1.7	19	30.6
Christian	8	6.8	5	8.1
Hindu	0	0.0	2	3.2
Non-denominational	6	5.1	3	4.8
Spiritual but not religious	44	37.6	22	35.5
Other	13	11.1	5	8.1
**Origin of birth** ^a^ (*χ*^2^ = 14.0, ***P* = .02**)	*n*	*%*	*n*	*%*
Africa	4	3.4	0	0.0
Asia	3	2.6	6	9.7
Europe	92	78.6	37	59.7
North America	13	11.1	16	25.8
South America	2	1.7	2	3.2
Oceania	2	1.7	1	1.6
**Psychiatric diagnoses**	*n*	*%*	*n*	*%*
Not disclosed (*χ*^2^ = 0.49, *P* = .49)	4	3.4	1	1.6
Severe depression (*χ*^2^ = 2.16, *P* = .14)	21	17.9	6	9.7
Bipolar (*χ*^2^ = 0.03, *P* = .86)	5	4.3	3	4.8
Psychosis (*χ*^2^ = 0.21, *P* = .65)	1	0.9	1	1.6
Anxiety disorder (*χ*^2^ = 0.85, *P* = .36)	17	14.5	6	9.7

^a^“In which country were you brought up? That is, in which country did you spend most of your childhood?,” written response. The responses were grouped by continent to allow for statistical analysis.

*t* = Student *t-*test value; *χ*^2^ = chi square test value; *U* = Mann–Whitney U test value.

Although the participants did not differ in their history of psychiatric diagnoses, psychedelic respondents exhibited significantly higher ED traits in the ESP than meditation respondents (presented in Table 3). Of psychedelic respondents, 23 (20%) reported suffering or having suffered from an ED in the past, while only 4 (6%) of meditation respondents reported this (*χ*^2^ = 5.52, *P* = .019).

**Table 3:** ED traits of the participants.

	Psychedelics (*n* = 117)				Meditation (*n* = 62)			
	*M*	*SD*	*n*	*%*	*M*	*SD*	*n*	*%*
**ESP** (*t* = 3.13, ***P* = .002**)	1.26	1.18			0.73	0.85		
**ESP ≥ 2** ^a^ (*χ*^2^ = 5.76, ***P* = .016**)			43	36.8			12	19.4

^a^Participants above cut-off, representing heightened ED traits.

*t* = Student *t-*test value; *χ*^2^ = chi square test value.

The participants’ psychedelic use and meditation habits are presented in Table 4. Psychedelic respondents had more psychedelic use history and practiced less meditation than meditation respondents. Most meditation respondents were long-term practitioners (over 5 years) and practiced daily. Over half (53%) were psychedelic-naive, while 13% had used classical psychedelics within the last year.

**Table 4:** Psychedelic use and meditation habits of the participants.

	Psychedelic (*n* = 117)		Meditation (*n* = 62)	
Classical psychedelic use history ^a^ (*U* = 1327.0, *P* < .001)	*n*	*%*	*n*	*%*
Never	1	0.9	33	53.2
Once	9	7.7	3	4.8
2–5 times	22	18.8	11	17.7
6–10 times	23	19.7	4	6.5
10–50 times	46	39.3	10	16.1
Over 50 times	16	13.7	1	1.6
**Time since last classical psychedelic** (*U* = 1277.5, ***P* < .001**)	*n*	*%*	*n*	*%*
Over a year ago or never	28	23.9	54	87.1
6–12 months ago	17	14.5	3	4.8
3–6 months ago	11	9.4	1	1.6
1–3 months ago	19	16.2	0	0.0
Less than 1 month ago	42	35.9	4	6.5
**Meditation practice** (*U* = 872.0, ***P* < .001**)	*n*	*%*	*n*	*%*
Never	45	38.5	0	0.0
Few times per year	10	8.5	2	3.2
Monthly	23	19.7	0	0.0
Weekly	21	17.9	12	19.4
Daily	15	12.8	36	58.1
Several times a day	3	2.6	12	19.4
**Meditation history** (*U* = 1664.5, ***P* < .001**)	*n*	*%*	*n*	*%*
None	34	29.1	0	0.0
Less than a year	10	8.5	3	4.8
1–2 years	15	12.8	1	1.6
2–5 years	15	12.8	4	6.5
Over 5 years	43	36.8	54	87.1

^a^“Have you ever tried classical psychedelics (e.g., LSD, psilocybin / ‘magic mushrooms’, ayahuasca, DMT, 5-MeO-DMT)?,” answered with multiple choice.

*U* = Mann–Whitney U test value.

### Descriptive data of the psychedelic and meditation experiences

Details of the drugs and meditation styles used to facilitate the Experience are presented in Table 5. Of psychedelic respondents, 69% reported an experience with classical psychedelics only, most often psilocybin (51% of classical only reports) and/or LSD (32%); 21% reported combining classical and non-classical psychedelics, most commonly cannabis (79% of polydrug reports), but also MDMA (38%) and ketamine (17%); and 10% reported an experience with non-classical psychedelics only, MDMA (75% of non-classical psychedelics) or cannabis (25%).

**Table 5:** Psychedelics and meditation styles used to facilitate the experience

Individual psychedelics used	*n*	*%*
LSD or analogues	40	34.2
Psilocybin or ‘magic mushrooms’	56	47.9
Ayahuasca	8	6.8
N,N-DMT	5	4.3
5-MeO-DMT or Bufo Alvarius	7	6.0
Mescaline or psychoactive cacti	2	1.7
MDMA, ‘ecstasy’ or ‘molly’	18	15.4
Ketamine	4	3.4
Cannabis	22	18.8
*S. divinorum*	1	0.9
**Aggregated drug groups**	*n*	*%*
Classical psychedelics only^a^	81	69.2
Non-classical psychedelics only^b^	12	10.3
Polydrug with classical and non-classical	24	20.5
**Meditation**	*n*	*%*
Silent meditation, mindfulness	49	79.0
Voice meditation (e.g*.* chanting)	3	4.8
Breathwork (intentional manipulation of breath)	6	9.7
Bodywork (e.g*.* yoga)	3	4.8

The participant could select several*.*

^a^LSD or analogues, psilocybin, ayahuasca, N,N-DMT, 5-MeO-DMT, mescaline, and ibogaine.

^b^ *S. divinorum*, MDMA, ketamine, and cannabis.

Upon analysis, “mindfulness” and “silent sitting or lying down” were combined into a single variable because there was no meaningful difference between them and the participants used them interchangeably. Most meditation respondents reported experiences with these (79%), and a minority with breathwork, chanting, or yoga. The participants named a broad variety of traditions and styles, including vipassana, zen, dzogchen, anapanasati, and Transcendental Meditation. One meditator reported a spontaneous experience while not actively meditating.

Time since the Experience is presented in Table 6. Meditation respondents reported older experiences than psychedelic respondents, with 42% reporting an experience that took place over 5 years before.

**Table 6:** Time since the experience*.*

Time since the experience ^a^	Psychedelics (*n* = 117)				Meditation (*n* = 62)			
(*U* = 2961.5, *P* = .039)	*n*		*%*		*n*		*%*	
<2 weeks	5		4.3		2		3.2	
2–4 weeks	6		5.1		5		8.1	
1–3 months	15		12.8		3		4.8	
3–6 months	8		6.8		3		4.8	
6–12 months	11		9.4		2		3.2	
1–2 years	15		12.8		10		16.1	
2–5 years	29		24.8		11		17.7	
> 5 years	28		23.9		26		41.9	

^a^“When did the Experience happen?,” answered with multiple choice.

*U* = Mann–Whitney U test value.

### Descriptive data of perceived changes in body attitudes and current body appreciation

In general, participants self-assessed their body dissatisfaction to have reduced slightly, and their body appreciation and body connection to have increased slightly as a result of the Experience. Descriptive data of perceived body attitude changes and current body appreciation are presented in Table 7. Differences between psychedelic and meditation respondents were examined in the regression models.

**Table 7:** Perceived body attitude changes and current body appreciation.

	Psychedelics (*n* = 117)		Meditation (*n* = 62)	
Perceived body attitude change	*M*	*SD*	*M*	*SD*
Body dissatisfaction^a^	2.63	0.79	2.39	1.03
Body appreciation^b^	3.54	0.75	3.87	1.02
Body connection^c^	3.68	0.88	4.02	0.95
**Current body appreciation**	*M*	*SD*	*M*	*SD*
BAS-2	3.39	0.73	3.78	0.56

Perceived body attitude changes were collected with the question: “As a result of the Experience, did you notice any persisting increases or decreases in the following?,” 1 signifying ‘great decrease’, through 3 ‘no change’, and 5 ‘great increase’.

^a^“Your critical thoughts and feelings about your body.”

^b^“Your acceptance and appreciation for your body.”

^c^“Your sense of being ‘at home’ in your body.”

### Body image themes in the reports and inter-rater reliability

**Figure 1:**
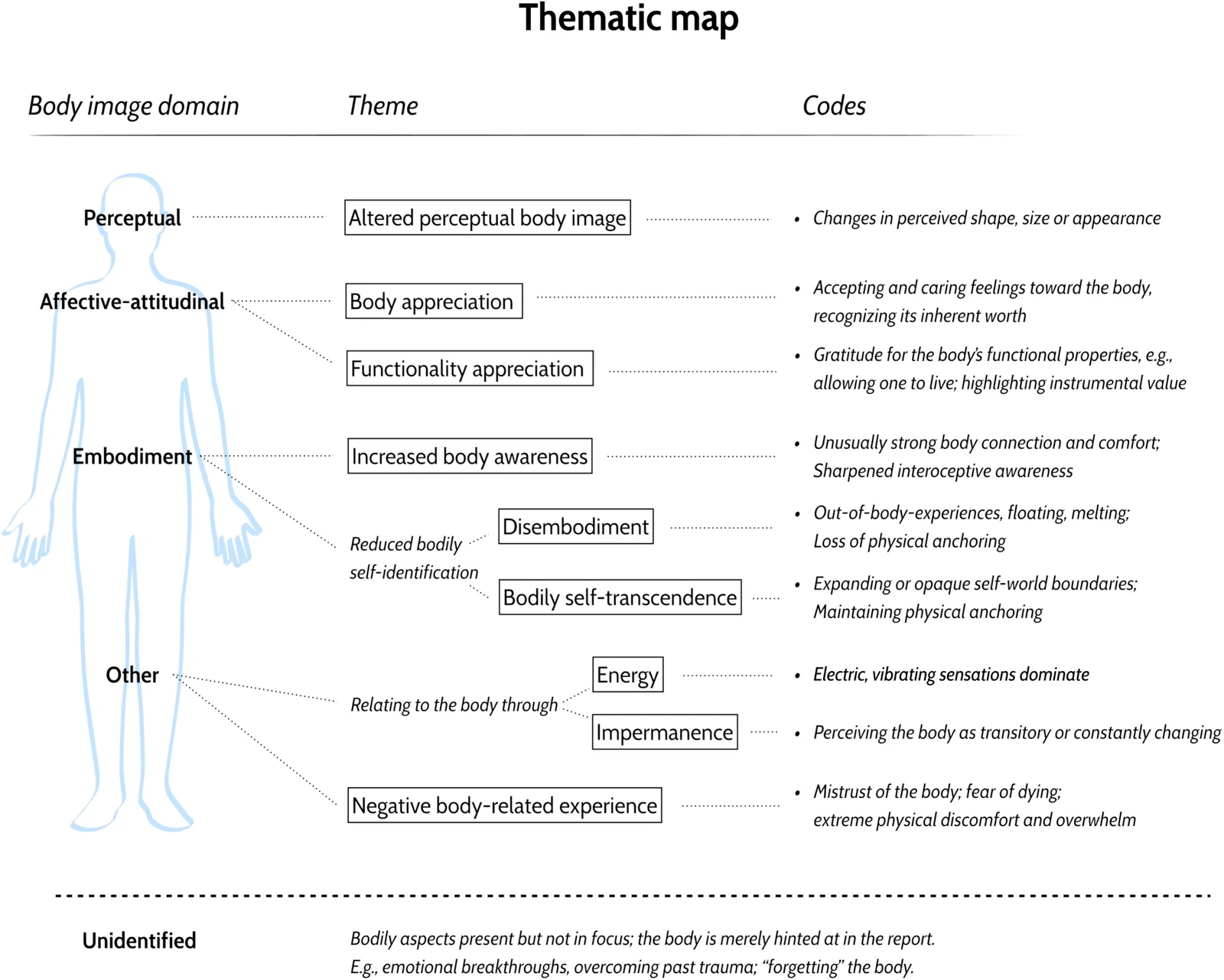
Thematic map of body image-related themes in reports of psychedelic and meditative experiences.

H.V. organized the thematic map of body image themes in reports of psychedelic and meditative experiences (*n* = 90 psychedelic, *n* = 48 meditation) by body image domain and phenomenological similarity, presented in Fig. 1. Here we describe all main themes and subthemes with examples from the participants. For a more comprehensive account of the themes, see Supplementary Material S2 (https://osf.io/whma3/).

The main theme ‘Altered Perceptual Body Image’ was defined as changes or updates in one’s “mental image” of their appearance, size, or shape. These ranged from detail-oriented (“My hands looked bigger,” female, 22, LSD) to holistic (“[I felt] light, beautiful, smaller, tinier, softer…,” female, 28, psilocybin). Some reported seeing their appearance in the mirror as changed. Some described novelty and astonishment in seeing themselves as fantastical creatures.

The main theme ‘Affective-Attitudinal Body Image’ was defined as feelings and attitudes toward one’s body’s appearance, functionality, or inherent worth. The reports were positively charged in this domain. The subtheme ‘Body Appreciation’ described holistic acceptance, care, and resolutions of self-judgment toward one’s body: “The deep appreciation and acceptance, the care I felt, was touching … All the judging, evaluating, pushing, and shaming melt away” (female, 29, metta, vipassana); “I felt like everything got the right proportions. I felt that I’m supposed to be like this” (female, 42, psilocybin). Some participants reported noticing new aspects about their body that they liked and paying less attention to the ones they did not like. Another subtheme in this domain, ‘Functionality Appreciation’, was defined as the appreciative acknowledgement of the body’s ability to function and allow life: “I understand my body as a gift that provides the framework for allowing my consciousness to be present/embodied in this human world” (female, 64, shamanic sound work).

The main theme ‘Embodiment’ described experiences of altered body awareness and connection. Its subtheme ‘Increased Body Awareness’ described a heightened sense of embodiment, body connection, and sensitivity to bodily sensations: “I remember feeling more at home in my body, feeling much more connecting [sic] to the world in an embodied and tactile way” (female, 39, MDMA); “[My] bodily sensations became somehow more sharp and vivid” (male, 28, psilocybin). The body was described in terms of being open, co-operative, and unusually alive: “I had a sense of the entire body being extraordinarily alive, and that the experience of walking was strongly present and energetic” (female, 41, zazen).

The Embodiment subthemes ‘Disembodiment’ and ‘Bodily Self-Transcendence’ encompassed different types of reduced bodily self-identification, separated on the basis on having or not having embodied presence. Disembodiment described intense experiences in which embodied presence was lost, defined in the codebook as “a disconnected body experience where one’s sense of body awareness, ownership, and/or self-location is disrupted, such as floating, out-of-body-experiences (OBE), or not having a body”. These narratives had features of oceanic boundlessness, OBEs, and such strong depersonalization that the body was perceived as alien. Although such phenomena could, in principle, be differentiated, these experiences were often described in brief, metaphorical statements that lacked sufficient detail for finer-grained distinction without interpretation: “I left my body there on the mattress” (other, 33, ayahuasca); “For parts of the experience I was completely oblivious to my physical body. In other parts, it felt alien, spread over more dimensions than ever before” (male, 37, DMT). Perceptions of melting and floating were common: “My proprioception disappeared completely, and I was a consciousness floating in a void” (male, 46, psilocybin). Some described sensory sensitivity remaining without bodily form: “The sense of body disappeared, some moments there were more like, just sensations without the body, merging with the sensations” (female, 29, LSD);

As [my body] ‘melted’ I scanned down into my feet, and I couldn’t distinguish a context, my feet were pure sensation and they felt as if they had dissolved. As I scanned upwards more of the confines of my body dissolved. I went from feeling like I was the one breathing to feeling like I was BEING Breathed. … I felt my face disappear and for several minutes I lost the ability to know myself in the context of my body. After a few minutes I felt myself resolidify from head to toe. Although I felt like I had a different body than before, with different solidity. (female, 54, vipassana)

In ‘Bodily Self-Transcendence’, embodied presence remained while body boundaries became translucent, shifted, or no longer contained one’s sense of self: “I felt very firmly rooted in my body but also like I was expanding outward into the universe” (female, 41, vipassana); “The boundary of my skin ceased to be the edge of ‘me’” (male, 27, dzogchen). These narratives recontextualized the self into a larger whole and described unity, but the experiences were physically anchored and integrative rather than disintegrative: “I felt no difference in touch between my skin, my friend’s skin and the air. It was all connected and encompassed in the same movement” (male, 50, LSD);

My body was doing things just like it had been always, but I didn't identify with it, it was more like a part of the whole world or environment, just like the road and the air and the trees outside when I was walking. (male, 48, zazen)

A recurring phrase in both Disembodiment and Bodily Self-Transcendence narratives was “a vessel,” used to differentiate the body from the self: “My body seemed just to be a vessel I am in at the moment, not the true me or in any way limiting my existence” (female, 43, silent meditation).

The main theme ‘Other’ included the subthemes ‘Body as Energy’ and ‘Impermanence’. In the former, the body was perceived through sensations of energy or vibration: “I was made aware of the bodies’ vibrational structure” (male, 53, mescaline). These narratives expressed high attunement of sensory flow and a reduced perception of solidity: “My body was pure sensation – it’s hard to explain, but [it] felt like pulsing energy” (female, 54, vipassana). Impermanence described time-related perceptions of the body as constantly changing and transitory: “[I] woke up to experiencing my entire mind body phenomenon changing on every moment” (male, 64, vipassana).

The theme ‘Negative Body-Related Experience’ included body-related negative feelings and challenges that could not be resolved within the experience, e.g. unsettling unfamiliarity and fears of losing bodily control or dying: “I was feeling extremely uncomfortable in my body. I couldn’t move” (female, 24, LSD, ketamine and cannabis).

Finally, ‘Unidentified’ encompassed cases from which no bodily themes could be identified at face value although the participant indicated their presence in the categorical thematic question. Many of these narratives resembled certain themes but were not specific enough to code without interpretation. For example, “oceanic boundlessness” was not coded as Disembodiment or Bodily Self-Transcendence, and emotional experiences were not coded as Increased Body Awareness unless the reports specified bodily aspects. The category ‘No bodily aspects’ represented the participants who categorically stated that bodily aspects were not present, encompassing the lack of bodily reference.

Measures of agreement on the presence of the themes in the reports are presented in Table 8. There was substantial agreement (0.64–0.83) on Altered Perceptual Body Image, Body Appreciation, Functionality Appreciation, Increased Body Awareness, and Disembodiment, and moderate agreement (0.49–0.58) on Bodily Self-Transcendence, Body as Energy, Impermanence, and Unidentified. Agreement on Negative Body-related Experience was fair (0.35). The codebook included a miscellaneous theme ‘Other: Other’, but it was omitted from further analysis because of nonexistent agreement (−0.03). Discrepant codings (*n* = 62) were settled in a consensus coding meeting.

**Table 8:** Inter-rater agreement on the presence of themes.

	kappa	*P*
**Altered perceptual body image**	.75	<.001
**Affective-attitudinal body image**		
Body appreciation	.83	<.001
Functionality appreciation	.79	<.001
Pooled affective-attitudinal^a^	.81	<.001
**Embodiment**		
Increased body awareness	.72	<.001
Disembodiment	.64	<.001
Bodily self-transcendence	.50	<.001
Pooled embodiment^a^	.62	<.001
**Other**		
Body as energy	.58	<.001
Impermanence	.53	<.001
Other: Other	−.03	.699
Pooled other^b^	.66	<.001
**Negative body-related experience**	.35	<.001
**Unidentified**	.49	<.001

^a^The pooled rating indicates if any of the subthemes within the thematic category was rated as occurring.

^b^“Pooled other” did not include "Other: Other,” since it was not reliable.

### Occurrences of body image themes in the reports

Table 9 presents the themes’ occurrences in the psychedelic and meditation reports. Almost half of all psychedelic and meditation reports included embodiment-related themes. In the psychedelic reports, Disembodiment was most common (27%), closely followed by Increased Body Awareness (25%). Disembodiment showed a modestly but significantly greater frequency in psychedelic reports compared to meditation reports, while Increased Body Awareness did not differ significantly between these.

**Table 9:** Theme occurrences in the psychedelic and meditation reports.

Theme	Psychedelics (*n* = 117)		Meditation (*n* = 62)				
	*n*	*%*	*n*	*%*	*χ* ^2^	*P*	*V*
**Altered perceptual body image** ^a^	10	8.5	0	0.0	5.61	**.018**	**0.18**
**Affective-attitudinal body image** ^a^							
Body appreciation^b^	10	8.5	1	1.6	3.38	.066	0.14
Functionality appreciation^b^	7	6.0	3	4.8	0.10	.751	0.02
Pooled affective-attitudinal^c^	15	12.8	4	6.5	1.73	.188	0.10
**Embodiment** ^a^							
Increased body awareness^b^	29	24.8	9	14.5	2.56	.110	0.12
Disembodiment^b^	32	27.4	8	12.9	4.87	**.027**	**0.17**
Bodily self-transcendence^b^	6	5.1	16	25.8	16.10	**<.001**	**0.30**
Pooled embodiment^c^	57	48.7	27	43.5	0.44	.510	0.05
**Other^b^**							
Body as energy^b^	8	6.8	11	17.7	5.08	**.024**	**0.17**
Impermanence^b^	0	0.0	4	6.5	7.72	**.005**	**0.21**
Pooled other^c^	8	6.8	14	22.6	9.32	**.002**	**0.23**
**Negative body-related experience^b^**	4	3.4	0	0.0	2.17	.141	0.11
**Unidentified^d^**	20	17.1	9	14.5	0.20	.656	0.03
**No bodily aspects^e^**	27	23.1	14	22.6	0.01	.940	0.01

^a^Predefined themes.

^b^Data-driven themes.

^c^The pooled rating indicates if any of the subthemes within the thematic category was rated as occurring.

^d^“Unidentified”= participants categorically stating that bodily aspects were present but none could be identified from the reports.

^e^“No bodily aspects” = participants categorically stating that bodily aspects were not present.

*χ* ^2^ = chi square test value; *V* = Cramer’s *V* test value. Values in bold represent statistically significant differences between psychedelic and meditation reports (*p* < .05).

In the meditation reports, the most common theme was Bodily Self-Transcendence (26%), followed by Body as Energy (18%). Both themes were significantly more frequent in the meditation reports, with Bodily Self-Transcendence showing the most substantial difference among all identified themes.

Three themes were exclusive to either psychedelic or meditation reports with smaller occurrences. Altered Perceptual Body Image was only present in psychedelic reports (9%), all cases involving classical psychedelics. Impermanence only occurred in meditation reports (7%), all cases involving vipassana practices. Negative Body-Related Experience only occurred in psychedelic reports (3%), two cases involving heavy polydrug use, one, a classical psychedelic, and one, cannabis only.

Table 10 breaks down the themes’ occurrences by drug type in psychedelic reports: classical psychedelics, MDMA, and polydrug combining classical and non-classical psychedelics. Increased Body Awareness was significantly more common in MDMA reports than classical psychedelic or polydrug reports with a medium to large effect size. The theme Body Appreciation showed a small to medium effect favoring MDMA reports over classical psychedelic or polydrug reports. All reports with this theme involved either psilocybin, LSD, MDMA, or their combination.

**Table 10:** Theme occurrences in the psychedelic reports by drug group.

	Classical ^a^ (*n* = 81)		MDMA ^b^ (*n* = 9)		Polydrug ^c^ (*n* = 24)			
	*n*	*%*	*n*	*%*	*n*	*%*	*P*	*V*
**Altered perceptual body image**	9	11.1	0	0.0	1	4.2	.537	0.13
**Affective-attitudinal body image**								
Body appreciation	5	6.2	3	33.3	2	8.3	**.029**	**0.26**
Functionality appreciation	5	6.2	0	0.0	2	8.3	.811	0.08
Pooled affective-attitudinal^d^	10	12.3	3	33.3	2	8.3	.152	0.18
**Embodiment**								
Increased body awareness	16	19.8	7	77.8	6	25.0	**.002**	**0.36**
Disembodiment	22	27.2	2	22.2	7	29.2	1.000	0.04
Bodily self-transcendence	6	7.4	0	0.0	0	0.0	.462	0.15
Pooled embodiment^d^	37	45.7	7	77.8	12	50.0	.201	0.17
**Other**								
Body as energy	6	7.4	0	0.0	2	8.3	1.000	0.08
Impermanence	0	0.0	0	0.0	0	0.0	n/a	n/a
Pooled other^d^	6	7.4	0	0.0	2	8.3	1.000	0.08
**Negative body-related experience**	1	1.2	0	0.0	2	8.3	.201	0.19
**Unidentified**	15	18.5	1	11.1	4	16.7	1.000	0.05
**No bodily aspects**	21	25.9	1	11.1	3	12.5	.381	0.15

^a^Reports of classical psychedelics only.

^b^Reports of MDMA only.

^c^Reports of polydrug use with classical and non-classical psychedelics.

^d^The pooled rating indicates if any of the subthemes within the thematic category was rated as occurring.

*P =* Fisher’s exact test value; *V* = Cramer’s *V* test value. Values in bold represent statistically significant differences between drug types (*p* < .05).

### Eating disorder traits in relation to body image themes in the reports

Nineteen separate binary logistic regression models were performed with each dummy-coded theme in either psychedelic or meditation reports as the dependent variable and ED traits (ESP score) as the independent variable. The models were adjusted for age and sex (binary).

The models with Body Appreciation and Functionality Appreciation in psychedelic reports as dependent variables were statistically significant (*χ*^2^(3, 116) = 10.598, *P* = .014, and *χ*^2^(3, 116) = 8.534, *P* = .036, respectively) and fit the data (Hosmer-Lemeshow *χ*^2^ = 6.251, *P* = .619; *χ*^2^ = 12.470, *P* = .131), explaining 19.7% of the variance in Body Appreciation, and 19.4% of Functionality Appreciation in psychedelic reports (Nagelkerke *R^2^*). Each one-point increase in the ESP score more than doubled the odds of Body Appreciation (*B* = 0.774, *P* = .009, *OR* = 2.169) and Functionality Appreciation in psychedelic reports (*B* = 0.903, *P* = .012, *OR* = 2.467).

The model with Body Appreciation in meditation reports as the dependent variable was statistically significant *χ*^2^(3, 59) = 10.138, *P* = .017. However, the meditation reports had only one case of Body Appreciation, reported by a participant with an ED history according to ESP item 4. Thus, the model could not be interpreted in a meaningful way and should be viewed as purely descriptive.

The model with Increased Body Awareness in meditation reports as the dependent variable was statistically significant *χ*^2^(3, 59) = 10.480, *P* = .015. The model fit the data (Hosmer-Lemeshow *χ*^2^ = 3.032, *P* = .932) and explained 29.7% (Nagelkerke *R*^2^) of the variance in Increased Body Awareness in meditation reports; however, ED traits were not significant. Younger participants and women were more likely to report the Increased Body Awareness theme than older individuals (*B* = −0.099, *P* = .017, *OR* = 0.906) and men (*B* = −2.199, *P* = .042, *OR* = 0.111). None of the other models were statistically significant (*P*s > .081). Details of non-significant models are listed in Supplementary Material S3 (https://osf.io/whma3/).

### Perceived body attitude changes in relation to body image themes in the reports and eating disorder traits

Three separate ordinal logistic regression models were performed with perceived changes in body dissatisfaction, body appreciation, and body connection (5-point Likert scale) as the dependent variables. Independent variables included all 11 dummy-coded themes, experience type (psychedelic/meditation), and ED traits (ESP score). The models were adjusted for age and sex (binary). Multicollinearity was low overall (variance inflation factors [VIFs] <2) and slightly elevated for Unidentified and No Bodily Aspects (VIFs 2.45–2.77).

The model with perceived changes in body appreciation as the dependent variable was statistically significant (*χ*^2^(15, *N* = 175) = 33.49, *P* = .004) with meditation respondents estimating greater body appreciation increases (*B* = 0.78, *OR* = 2.18, 95% CI [1.005, 4.733], *P* = .049).

The model with perceived changes in body connection as the dependent variable was significant (*χ*^2^(15, *N* = 175) = 47.40, *P* < .001). Affective-Attitudinal Body Image subthemes were associated with greater perceived body connection increases: Body Appreciation (*B* = 1.60, *OR* = 4.95, 95% CI [1.31, 18.70], *P* = .018) and Functionality Appreciation (*B* = 1.71, *OR* = 5.55, 95% CI [1.29, 23.85], *P* = .021). Embodiment subthemes Disembodiment and Increased Body Awareness were significant (*B* = 1.23, *OR* = 3.42, 95% CI [1.34, 8.72], *P* = .010, and *B* = 1.05, *OR* = 2.87, 95% CI [1.04, 7.90], *P* = .041, respectively), as was Body as Energy (*B* = 1.18, *OR* = 3.24, 95% CI [1.07, 9.83], *P* = .038). Meditation respondents reported greater increases in body connection than psychedelic respondents (*B* = 0.82, *OR* = 2.28, 95% CI [1.07, 4.87], *P* = .033), and men reported smaller increases than women (*B* = −0.89, *OR* = 0.41, 95% CI [0.22, 0.77], *P* = .006).

Also the model with perceived changes in body dissatisfaction as the dependent variable was significant (*χ*^2^(15, *N* = 175) = 36.29, *P* = .002). Disembodiment was associated with greater perceived reductions in body dissatisfaction (*B* = −1.56, *OR* = 0.21, 95% CI [0.09, 0.51], *P* < .001), as was Increased Body Awareness (*B* = −0.96, *OR* = 0.38, 95% CI [0.147, 0.997], *P* = .049). Again, meditation respondents estimated greater body dissatisfaction reductions (*B* = −0.89, *OR* = 0.41, 95% CI [0.19, 0.91], *P* = .028), particularly when reporting Impermanence (*B* = −2.31, *OR* = 0.10, 95% CI [0.01, 0.84], *P* = .034). Also participants with ED traits estimated greater reductions in body dissatisfaction (*B* = −0.30, *OR* = 0.74, 95% CI [0.55, 0.99], *P* = .045).

### Body image themes in the reports in relation to current body appreciation

We performed a linear regression model with BAS-2 as the dependent variable and all the 11 themes (occurrence 1/0), experience type (psychedelics/meditation), and ED traits (ESP score) as independent variables. The model was adjusted for age and sex (binary). Multicollinearity was low overall (VIFs < 2) yet slightly elevated for Unidentified and No Bodily Aspects (VIFs 2.45–2.77).

The model was statistically significant [*F*(15, 159) = 4.550, *P* < .001] and explained 30% of the variance in BAS-2 scores (*R*^2^ = 0.30, Adjusted *R*^2^ = 0.23). Meditation respondents had significantly higher body appreciation (*B* = 0.289, 95% CI [0.046, 0.533], *β* = 0.412, *t* = 2.348, *P* = .020), and those with ED traits had significantly lower body appreciation (*B* = −0.297, 95% CI [−0.391,−0.203], *β* = −0.469, *t* = −6.221, *P* < .001). No themes were associated with current body appreciation (*P*s > .128).

## Discussion

This study identified body image-related themes in retrospective reports of personally meaningful psychedelic and meditative experiences. We examined associations between body image themes in the reports, perceived changes in body attitudes (body dissatisfaction, body appreciation, and body connection), and current body appreciation, and whether participants’ current ED traits were associated with distinctive themes or changes in body attitudes. We compared psychedelic reports with meditation reports, and reports of classical psychedelics, MDMA, and polydrug use with classical and non-classical psychedelics, with each other.

When asked to recall their “most personally meaningful or important” experience, most participants chose an experience from over a year before, with over 40% of meditation respondents reporting an experience from over 5 years before. While demonstrating the subjective importance of these experiences, this also necessitates a reminder that the reports reflect memories which have been narratively integrated a long time ago and differ from the experiences they are about. Many aspects of ASCs are state-specific and inaccessible in retrospect. Details and timelines are forgotten over time and distorted by salience, interpretative reconstruction, and meaning-making (Cardeña and Pekala 2014, Moreira-Almeida and Lotufo-Neto 2017). What is retained is shaped by its most intense and final moments, reflecting a broader divergence between the experiencing and the remembering self (Kahneman and Riis 2005). Thus, the themes in the reports should be viewed as narrative body image features that are prompted by ASCs but separated from them.

The psychedelic and meditative narratives similarly conveyed heightened sensitivity to sensory flow with a reduced perception of the body as a solid, bounded entity. The main difference was in embodied presence, which meditators were more likely to maintain throughout. The most common theme in psychedelic reports was Disembodiment, describing intense and ineffable disintegrative bodily experiences where participants could no longer perceive themselves as being “in” or “as” their body. While this was also present in some meditation reports, these most commonly described vanishing or expanding body boundaries without the loss of embodied presence, here named Bodily Self-Transcendence.

This broadly aligns with factor-analytic ASC studies. Our definition of Disembodiment was based on the disembodiment factor in the Altered States of Consciousness Rating Scale (5D-ASC/11-ASC; Studerus et al. 2010), with items referring to floating, OBEs, and not having a body, i.e*.* disruptions in overall body awareness or aspects of the bodily self, such as self-location, ownership, or first-person perspective (Blanke 2012). We differentiated Bodily Self-Transcendence from Disembodiment based on embodied presence, which made these narratives qualitatively different, and formulated the theme in reference to Berkovich-Ohana et al. (2013), suggesting that the loss of body boundaries occurs along a gradient depending on the intensity of the ASC in meditation.

In the 5D-ASC/11-ASC framework, disembodiment is one subscale of oceanic boundlessness whose core factor is the experience of unity. However, the disembodiment items also load on the dread of ego dissolution dimension, reflecting the intensity and uncontrollability of the experience (Studerus et al. 2010). Dose–response studies show that oceanic boundlessness intensifies with dose for classical psychedelics, yet the dose-dependence of disembodiment specifically is less consistent (Hirschfeld and Schmidt 2021, Hirschfeld et al. 2023). While meditation studies using this framework are very limited, disembodiment and unity have been linked to practice intensity and to each other, with qualitative descriptions of self-transcendent boundary dissolution similar to those in our study (Galante et al. 2024). Thus, the Bodily Self-Transcendence theme likely maps onto lower-level disembodiment according to the 5D-ASC/11-ASC.

In the Disembodiment narratives, losing integrated body awareness did not always signify losing awareness of interoceptive sensations. Rather, these were described vibrantly and with flexible meanings, e.g*.* “pure sensation” and “life force”. A theme of experiencing the body through sensations of flowing energy emerged. In the psychedelic reports, this was mainly described with Disembodiment, while in the meditation reports, this was the second most common theme and also occurred in integrated embodiment narratives. Strong sensory attunement with heightened embodied presence was almost as common as Disembodiment in the psychedelic reports, particularly of MDMA, yet these reports did not highlight stimulating vibrations or reduced solidity.

Moreover, the themes Altered Perceptual Body Image and Negative Body-Related Experience were exclusive to psychedelic reports and Impermanence to meditation reports. These differences raised a question whether theoretical contamination makes psychedelic and meditative phenomenology appear more similar than they are, as the discourses share terminology. The psychedelic reports seemed to describe more abrupt and bottom-up flooded bodily experiences, which is reasonable considering the fast-acting nature of drugs compared to slow-paced and effort-consuming meditation practices. A previous sentiment analysis of the open reports similarly showed the psychedelic reports to have more extreme positive and negative emotions compared to the meditation reports, although their semantics and insight content were very much alike (Jylkkä et al. 2025, Kallio-Mannila et al. 2025).

Different contexts can also shape interpretations very distinctively. Many meditators reported experiences from retreats and expressed devotion to specific traditions, whereas psychedelic narratives echoed a variety of settings and motivations from couples’ time through ceremonial work to parties. These contexts cannot be separated from how the bodily self-experiences were observed and assigned meaning. For example, impermanence is a core Buddhist tenet and was only reported as a body image theme by vipassana practitioners, and the notion of body as energy is explicitly discussed in Goenka’s vipassana and some other yogic traditions.

Psychedelic and meditation respondents also exhibited certain differences that warrant caution to pairwise comparisons. Meditation respondents were older, reported long-term daily practice, had much lower ED traits, very high current body appreciation, and gave higher ratings in all perceived body attitude changes. While there were no differences in histories of psychiatric diagnoses, more psychedelic respondents self-reported ED histories, implying more serious self-image problems and vulnerabilities that may remain subclinical or undiagnosed. Such aspects can certainly be reflected on the participants’ typical bodily self-experience, what they seek, consider salient, and report. For example, the relative stability of embodied presence in meditation reports could, in theory, have links to underlying differences in, e.g*.* interoceptive awareness, affective stability, or other psychological factors.

The participants self-assessed perceived changes in body attitudes after writing the reports. These were not objective measures in relation to actual experiences, but they illustrated how subjectively impactful the participants considered these experiences for their body attitudes.

Participants reporting the Embodiment subthemes Increased Body Awareness and Disembodiment showed higher perceived reductions in body dissatisfaction, i.e. critical thoughts and feelings about the body, as did those reporting Impermanence. Reporting any positive Affective-Attitudinal Body Image subtheme, such as finding acceptance for the body or feeling gratitude for its functional properties (Body and Functionality Appreciation), was associated with perceived increases in body connection, i.e. feeling more “at home” in the body. Those describing Increased Body Awareness, Disembodiment, and Body as Energy, also gave more increased body connection ratings.

Thus, embodiment-related narratives were broadly associated with perceived reductions in negative body affect and improvements in embodiment quality, and in turn, narratives with reduced negative body affect were associated with perceived improvements in embodiment quality. No themes, however, were associated with perceived increases in body appreciation or current body appreciation, implying that the body image-related narrative content was primarily linked with perceived relief from negative beliefs and obstacles to uncomplicated embodiment rather than introducing explicitly positive beliefs.

While the link between Increased Body Awareness and perceived body attitude improvements seems intuitive, it is interesting that Disembodiment was also associated with perceived reductions in body dissatisfaction. Several reports with this theme reframed the body as “a vessel” and expressed that the body was no longer seen as a defining feature of self, but rather, a functional part that allowed participants to experience life. It seems plausible that such a perspective could promote less preoccupied and more functionally oriented body attitudes in alignment with notions from Peck et al. (2024).

Theoretically, disembodiment has also been linked to increases in body-related attunement, and it has been suggested that arresting rigid top-down control may allow for body-related multisensory information to be accessed more directly, promoting flexibility in embodiment and body attitudes (Riva 2018). In support of this, some clinical observations have linked ketamine-induced disembodiment to enhanced somatic awareness and interaffectivity in individuals with compromised embodiment (Sarasso et al. 2024). However, in this study’s context, this remains a hypothetical notion rather than an explanation.

Current ED traits, including a lifetime history of ED, correlated with reporting positive affective-attitudinal body image themes and perceived reductions in body dissatisfaction. It is important to remember that these associations are cross-sectional and that ED traits at the time of the experience are not known. Individuals with active body image concerns are likely more incentivized to report warm body-related feelings as personally meaningful, whereas those with no such issues would focus on other aspects. Facing personally challenging themes is common in psychedelic and meditation experiences, and evidence suggests that experiential acceptance and adaptive reappraisal can help their processing (Lindahl et al. 2017, Wood et al. 2024). Several Body Appreciation narratives expressed long-standing body image concerns, relief over finding acceptance for the body, and a motivation for maintaining harmony with it.

Empathy enhancement may have played a role in these narratives. The Body Appreciation theme only occurred in reports of MDMA, psilocybin, and LSD, either alone or in combination, and previous studies link these substances with increased affective empathy (Preller and Vollenweider 2019). Interestingly, in the one meditation report describing Body Appreciation, the participant—who also reported an ED history—was practicing metta (loving-kindness meditation) toward the end of a vipassana retreat. Recalling and reporting such memories of unusual self-compassion could plausibly encourage a perception of being more “at home” in one’s body.

Although the task of recalling the most “personally meaningful or important experience” caused the reports to be predominantly positive, some psychedelic reports also included frightening and negative body-related experiences. These illustrated how sudden, drastic reductions in the sense of predictability, paired with sensory acuity, can become overwhelming and result in perceiving the body as unfamiliar and unreliable. It needs to be acknowledged that both psychedelics and intense meditation can induce dissociative symptoms that persist beyond the acute experience and maladaptively affect embodiment and well-being, such as in depersonalization and derealization (Lindahl et al. 2017, Evans et al. 2023). It is crucial to develop support and integration practices, because while some individuals with body image problems report benefiting from these experiences, others may be vulnerable to destabilization.

### Limitations

This study has many limitations. Again, it needs to be highlighted that we studied memories, several of which were about events that happened over one or more years before. Retrospective reports are subject to the descriptive abilities of the participants, inevitable memory decay, and several sources of distortion, such as interpretative reconstruction, cultural framing, and situational factors (Cardeña and Pekala 2014, Moreira-Almeida and Lotufo-Neto 2017). These findings reflect meaning-making and do not generalize to real-time ASCs.

We based the level of analysis on available information and sample size. Several reports were condensed in brief, metaphorical statements, reflecting the ineffability and abstractness of the experiences. This was particularly noticeable in the Disembodiment theme. A finer-grained mapping of specific embodiment-related alterations would have been ideal, but the data did not support this. In the similarly heterogeneous Altered Perceptual Body Image, novel sights in the mirror and changed percepts of shape and size were analyzed together because further division would have rendered these themes statistically meaningless. Higher resolution studies may require more structured approaches.

Because the surveys presented thematic questions about bodily aspects, connectedness/alienation, and insight experiences in a randomized order, roughly 2/3 of the participants wrote other thematic reports before reporting bodily aspects. Connectedness/alienation may have primed participants to observe embodiment through this lens and insights may have, e.g*.* directed focus away from bodily aspects altogether. Fatigue over writing several reports may have also caused participants to overlook details and write briefly.

Another limitation was sampling. In several comparisons, the sample sizes were small and uneven. As the participants were self-selected, psychedelic and meditation respondents showed some demographic and other differences that limit comparability and raise questions about underlying factors. The participants represented distinct naturalistic contexts, and despite some population-level overlaps, average personality factors and values likely differ between psychedelic users and devoted meditators. The demands of meditation practice could, e.g. rule out individuals with more psychological vulnerabilities, who, in turn, could be more drawn toward substances.

ED traits were chosen to capture variance in body image-related rigidity and concern; however, other measures would have also been relevant. For example, interoceptive awareness, body dysmorphic beliefs, and other non-weight-based sources of body dissatisfaction were not captured by this choice. Moreover, our quantitative measures emphasized affective-attitudinal body image, whereas embodiment was the most prominent in the qualitative reports and deserves more targeted examination.

Methodologically, this study’s cross-sectional design rules out causal conclusions. While the participants reported body attitude changes, baselines are not known. Self-report measures always pose risks of bias, especially when answered retrospectively. Social desirability and demand characteristics were sources of bias, and the main task introduced positivity bias. The variability of facilitation methods could also be considered a confounder. It is also important to note that several of this study’s key *P*-values fall between .01 and .05. Individual findings should thus be treated as provisional and pending replication.

Finally, language is inseparable from narratively integrated experiences. Some differences in the reports may be explained by more meditators practicing Buddhism and more psychedelic respondents identifying as atheist. Contextual language may cause similar phenomena to appear different, and in turn, theoretical contamination may cause different phenomena to appear similar. Had we used quantitative instruments and not TA to examine bodily self-experience, the themes Disembodiment and Bodily Self-Transcendence may have appeared as a single phenomenon; however, qualitatively, these emerged as distinct phenomena based on different embodiment characteristics. We suggest that this information is viewed as complementary rather than as contradictory.

### Conclusions

In the context of ASC, “body image” has historically referred to alterations in embodiment rather than narrative or attitudinal aspects. To our knowledge, this is the first study to examine body image-related themes through this lens in psychedelic and meditative experience narratives, compare these between facilitation methods, and examine their associations with body attitudes. While the psychedelic and the meditation reports similarly included heightened sensory flow and reduced perceptions of the body as a solid, bounded entity, differences were found. The psychedelic reports involved a more drastic curve in disembodiment, and the meditation reports expressed a more integrated body awareness when describing reduced solidity, dissolving boundaries, and unitary experiences. The findings suggest that memories of unusual embodiment experiences, particularly of disembodiment and heightened body awareness, are associated with perceived reductions in body dissatisfaction and improved body connection. In turn, memories of unusual emotional acceptance toward the body are associated with perceived improvements in body connection. Moreover, the results indicate that individuals with ED traits may consider these experiences beneficial for negative affective-attitudinal body image. Affective empathy enhancement could play a role. However, longitudinal studies tracking body image measures before and after psychedelic and meditative experiences would be needed to inform about causal effects, preferably in a randomized controlled trial setting.

## Supplementary Material

Supplementary_material_niag062

## Data Availability

Data are publicly available at the Open Science Framework (https://osf.io/whma3/) with the exception of the narrative reports, which have been retracted to protect participant anonymity.
