# Supplementary material for: Body image phenomenology in reports of psychedelic and meditative experiences: main themes and associations with body attitudes
Source: Neurosci Conscious. 2026 Sep 21;2026(1):niag062. doi: 10.1093/nc/niag062 (PMC13592169; doi:10.1093/nc/niag062)
Supplement: Supplementary file 1 [file supplementary_material_niag062.zip › README to Opendata.pdf]

READ ME to Open Data

Body image phenomenology in reports of psychedelic and meditative experiences (BodyPsyMed_OpenData)

The variables explained in chronological order according to the Open Data file.

Demographics

group: 0 psychedelics; 1 meditation

age: age (years)

gender_1f_2m_3neither: 1 female; 2 male; 3 other

educationlevel: “Please choose the highest education level that you have completed”; 1 Primary education; 2 Lower Secondary education; 3 Higher Secondary education (for example, high school diploma); 4 Vocational education; 6 University: Bachelor’s degree; 7 University: Master’s degree; 8 University: Doctoral degree; 999 Other.

incomelevel: “Please estimate your personal income compared to the average income in your current country of residence”; 1 Much below average; 2 Below average; 3 Average; 4 Above average; 5 Much above average.

countryofbirth: “In which country were you brought up? That is, in which country did you spend most of your childhood? (If you don't wish to disclose this information, you can write “NA”)”

englishproficiency: “Please rate your ability in writing English”; 1 Native-like; 2 Very good; 3 Good; 4 Functional; 5 Fair; 6 Poor; 7 Very poor.

religion: "How would you describe your religion?”; 1 Agnostic; 2 Atheist; 3 Buddhist; 4 Christian; 5 Hindu; 6 Jewish; 7 Muslim; 8 Non-denominational; 9 Spiritual but not religious; 999 Other.

medit_freq: “Do you practice meditation?”; 0 No; 1 I have tried once or twice but do not practice regularly; 2 Few times per year; 3 Few times per month; 4 Every week; 5 Daily; 6 Several times a day.

medit_howlong: “For how long have you practiced meditation?”; 0 I do not meditate; 1 Less than a month; 2 1-6 months; 3 7-12 months; 4 Between 1 and 2 years; 5 Between 2 and 5 years; 6 Over 5 years.

medit_type: “What type of meditation do you practice?”

medit_type_silent: 1 practices silent sitting or lying down; 0 does not practice silent sitting or lying down
medit_type_voice: 1 practices voicework (e.g. chanting); 0 does not practice voicework (e.g. chanting)
medit_type_breath: 1 practices breathwork (intentional manipulation of breath); 0 does not practice breathwork (intentional manipulation of breath)
medit_type_body: 1 practices bodywork (e.g. yoga); 0 does not practice bodywork (e.g. yoga)
medit_type_mindfulness: 1 practices mindfulness; 0 does not practice mindfulness

classicalPsych_frequency: “Have you ever tried classical psychedelics (e.g., LSD, psilocybin / "magic mushrooms", ayahuasca, DMT, 5-MeO-DMT)?”; 0 Never; 1 Once; 2”, Twice; 3 3-5 times; 4 6-10 times; 5 10-50 times; 6 Over 50 times.

TimeSinceLastClassicalPsych: “Please indicate when was the last time you used classical psychedelics (e.g., LSD, psilocybin / "magic mushrooms", ayahuasca, DMT, 5-MeO-DMT)”; 0 Never or over a year ago; 1 9-12 months ago; 2 6-9 months ago; 3 3-6 months ago; 4 1-3 months ago; 5 2-4 weeks ago; 6 One week ago; 7 Less than one week ago.

Psychiatric: “Have you ever been diagnosed by a medical professional with any of the following conditions?”

Psychiatric_No_disclosure: I don't wish to give this information 1/0
Psychiatric_severe_depression: Severe depression 1/0
Psychiatric_bipolar: Bipolar disorder 1/0
Psychiatric_psychosis: Psychosis or schizophrenia 1/0
Psychiatric_anxiety: Anxiety disorder 1/0
Psychiatric_ASD: Autism spectrum disorder / Asperger 1/0
Psychiatric_OCD: Obsessive-compulsive disorder
Psychiatric_ADHD: Attention deficit hyperactivity disorder (ADHD or ADD) 1/0

substance_use_problem: "If you use psychoactive substances (e.g., alcohol, nicotine, illicit drugs), please rate how your use of such substances affects you and your close ones"; 0 Very negatively; 1 Slightly negatively; 2 Neither negatively nor positively; 3 Slightly positively; 4 Very positively; 999 I do not use psychoactive substances.

Eating disorder traits: the Eating disorder screen for primary care (ESP)

ESP1_INV: "Are you satisfied with your eating patterns?" (inverted); 1 No; 0 Yes.
2 ESP: "Do you ever eat in secret?"; 0 No; 1 Yes.
3 ESP: "Does your weight affect the way you feel about yourself?"; 0 No; 1 Yes.
4 ESP: "Do you currently suffer with or have you ever suffered in the past with an eating disorder?"; 0 No; 1 Yes.

Background of The Experience: "Please think of one psychedelic experience that you consider as the most meaningful or important in your life, in good or bad. Henceforth, we refer to this experience as "The Experience", and ask you to evaluate that experience, and only that experience."

exp_(drug): "Which drug did you use to facilitate The Experience?" (Psychedelic survey only; selecting several substances was permitted)

exp_LSD: LSD or analogues 1/0
exp_psilocybin: Psilocybin or 'magic mushrooms' 1/0
exp_ayahuasca: Ayahuasca 1/0
exp_DMT: N,N-DMT
exp_5meoDMT: 5-MeO-DMT or Bufo Alvarius 1/0
exp_mescaline: Mescaline or psychoactive cacti 1/0
exp_ibogaine: Ibogaine 1/0
exp_salvia: Salvia divinorum 1/0
exp_mdma: MDMA, 'ecstasy' or 'molly' 1/0
exp_ketamine: Ketamine 1/0
exp_cannabis: Cannabis 1/0
other_drug_dummy: "Other drug, please specify" 1/0
exp_other_drug_spec: written response

exp_drug_dose: "How large was the dose?"; 0 Cannot say; 1 Very small"; 2 Smaller than typical; 3 Typical dose; 4 Larger than typical; 5 Very large dose.

exp_drug_intensity: "How intense was the overall effect of the drug?"; 0 Not at all; 1 Slightly; 2 Moderately; 3 Strong (equivalent in degree to other strong experiences in my life); 4 Extreme (stronger than any other experience in my life).

exp_drug_confidence: "How confident are you the drug was what it was supposed to be?"; 0 The drug may have been another substance; 1 The drug may have been spiked or cut with another substance; 2 Quite confident but can't guarantee (i.e. drug not tested, origin unknown; 3 Very confident (i.e. drug tested or origin known.

exp_medit: "Which meditation technique did you use to facilitate The Experience?" (Meditation survey only; selecting several techniques was permitted)

exp_medit_silent: Silent sitting or lying down 1/0
exp_medit_voice: Voicework (e.g., chanting) 1/0

exp_medit_breath: Breathwork (intentional manipulation of breath) 1/0
exp_medit_body: Bodywork (e.g., yoga) 1/0
exp_medit_mindfulness: Mindfulness 1/0
exp_meditation_technique_specific: specific technique; written response (optional)
exp_medit_drug: “Did you use a psychoactive substance in addition to the meditation technique?” 1/0

exp_when: “When did The Experience happen?”; 0 Less than one week ago; 1 One week ago; 2 2–4 weeks ago; 3 1–3 months ago; 4 3–6 months ago; 5 6–12 months ago; 6 1–2 years ago; 7 2–5 years ago; 8 Over 5 years ago.

exp_where: “Where did The Experience happen?”; 0 Home or home-like environment; 1 Friend's home or home-like environment; 2 Unknown person's home or home-like environment; 3 Other private space; 4 Nature; 5 Ceremonial space or retreat; 6 Space designed for therapeutic purpose; 7 Public gathering (e.g., festival; 8 Other public space.

exp_who: “Who was present during The Experience?”; 0 Alone; 1 One other person; 2 Several people, all familiar; 3 Several people, some or all unfamiliar.

exp_guide: “Was there a guide, facilitator, or therapist present during The Experience?”; 0 No; 1 Yes, one; 2 Yes, several.

exp_purpose: “What was the main purpose of The Experience?” (selecting several options was permitted)

exp_purpose_nospecific: No specific purpose / Cannot say 1/0
exp_purpose_relax_enjoy: To relax and enjoy 1/0
exp_purpose_spiritual: Spiritual practice 1/0
exp_purpose_therapeutic: Therapeutic work 1/0
exp_purpose_curiosity: Curiosity 1/0
exp_purpose_social: Social reasons 1/0
exp_purpose_selfimprovement: Self-improvement 1/0
exp_purpose_distraction: Distraction from problems or boredom 1/0
exp_purpose_other: Other specific purpose 1/0
exp_planned: “Was The Experience planned?”; 0 Not at all; 1 Somewhat; 2 Yes.
exp_expect: “Did you have prior knowledge about what to expect?”; 0 Not at all; 1 Somewhat; 2 Yes.
exp_drug_meditation: “Was there a meditation technique involved in addition to the drug?” (Psychedelic survey only); 1 Yes; 0 No.

exp_drug_medit_silent: The drug experience involved silent sitting or lying down 1/0
exp_drug_medit_voice: The drug experience involved voicework (e.g., chanting) 1/0
exp_drug_medit_breath: The drug experience involved breathwork (intentional manipulation of breath) 1/0
exp_drug_medit_body: The drug experience involved bodywork (e.g., yoga) 1/0
exp_drug_medit_mindfulness: The drug experience involved mindfulness 1/0

The thematic question

body_yes_no: “Did you perceive your body in a different way or have any personally meaningful thoughts, feelings or insights about it?”; 1 Yes; 2 No; 3 Don’t know.

Perceived changes in body attitudes

prepost: “As a result of the Experience, have you noticed any persisting increases or decreases in the following?”

prepost_bodydissat: Body dissatisfaction (“Your critical thoughts or feelings about your body”); 1 Decreased a lot; 2 Decreased a bit; 3 Not changed (stayed the same); 4 Increased a bit; 5 Increased a lot.

prepost_bodyappre: Body appreciation (“Your acceptance and appreciation for your body”); 1 Decreased a lot; 2 Decreased a bit; 3 Not changed (stayed the same); 4 Increased a bit; 5 Increased a lot.

prepost_embodiment: Body connection (“Your sense of being ‘at home’ in your body”); 1 Decreased a lot; 2 Decreased a bit; 3 Not changed (stayed the same); 4 Increased a bit; 5 Increased a lot.

Standardised questionnaires

The Mystical Experience Questionnaire 30 (MEQ30)

MEQ30_Q1_mystical: 1–5 (Likert scale)
MEQ30_Q2_mystical: 1–5 (Likert scale)
MEQ30_Q3_mystical: 1–5 (Likert scale)
MEQ30_Q4_mystical: 1–5 (Likert scale)
MEQ30_Q5_mystical: 1–5 (Likert scale)
MEQ30_Q6_mystical: 1–5 (Likert scale)
MEQ30_Q7_mystical: 1–5 (Likert scale)
MEQ30_Q8_mystical: 1–5 (Likert scale)
MEQ30_Q9_mystical: 1–5 (Likert scale)
MEQ30_Q10_mystical: 1–5 (Likert scale)
MEQ30_Q11_mystical: 1–5 (Likert scale)
MEQ30_Q12_mystical: 1–5 (Likert scale)
MEQ30_Q13_mystical: 1–5 (Likert scale)
MEQ30_Q14_mystical: 1–5 (Likert scale)
MEQ30_Q15_mystical: 1–5 (Likert scale)
MEQ30_Q16_positive_mood: 1–5 (Likert scale)
MEQ30_Q17_positive_mood: 1–5 (Likert scale)
MEQ30_Q18_positive_mood: 1–5 (Likert scale)
MEQ30_Q19_positive_mood: 1–5 (Likert scale)
MEQ30_Q20_positive_mood: 1–5 (Likert scale)
MEQ30_Q21_positive_mood: 1–5 (Likert scale)
MEQ30_Q22_transcendence: 1–5 (Likert scale)
MEQ30_Q23_transcendence: 1–5 (Likert scale)
MEQ30_Q24_transcendence: 1–5 (Likert scale)
MEQ30_Q25_transcendence: 1–5 (Likert scale)
MEQ30_Q26_transcendence: 1–5 (Likert scale)
MEQ30_Q27_transcendence: 1–5 (Likert scale)
MEQ30_Q28_ineffability: 1–5 (Likert scale)
MEQ30_Q29_ineffability: 1–5 (Likert scale)
MEQ30_Q30_ineffability: 1–5 (Likert scale)

MEQ30_sum: MEQ30 score (sum of all items). Please note that the current MEQ_sum includes values for the qualitative sample only (psy n = 90; med n = 48).

The Warwick-Edinburgh Mental Wellbeing Scale (WEMWBS)

WEMWBS_optimistic: 1–5 (Likert scale)
WEMWBS_useful: 1–5 (Likert scale)
WEMWBS_relaxed: 1–5 (Likert scale)
WEMWBS_dealing_w_problems: 1–5 (Likert scale)
WEMWBS_thinking_clearly: 1–5 (Likert scale)
WEMWBS_close_to_others: 1–5 (Likert scale)
WEMWBS_make_up_my_own_mind: 1–5 (Likert scale)

The Peace of Mind Scale (PoM)

PoMS_1: 1–5 (Likert scale)
PoMS_2: 1–5 (Likert scale)
PoMS_3: 1–5 (Likert scale)
PoMS_4: 1–5 (Likert scale)
PoMS_5_INV: 1–5 (Likert scale)
PoMS_6: 1–5 (Likert scale)

PoMS_7_INV: 1–5 (Likert scale)

The Body Appreciation Scale 2 (BAS-2)

BAS1: 1–5 (Likert scale)
BAS2: 1–5 (Likert scale)
BAS3: 1–5 (Likert scale)
BAS4: 1–5 (Likert scale)
BAS5: 1–5 (Likert scale)
BAS6: 1–5 (Likert scale)
BAS7: 1–5 (Likert scale)
BAS8: 1–5 (Likert scale)
BAS9: 1–5 (Likert scale)
BAS10: 1–5 (Likert scale)

Psy-Flex

psy_flex1: 1–5 (Likert scale)
psy_flex2: 1–5 (Likert scale)
psy_flex3: 1–5 (Likert scale)
psy_flex4: 1–5 (Likert scale)
psy_flex5: 1–5 (Likert scale)
psy_flex6: 1–5 (Likert scale)

userid: Participant id

The presence of body image-related themes in the reports

PBI: Altered Perceptual Body Image 1/0
ABI_B: Body Appreciation 1/0
ABI_F: Functionality Appreciation 1/0
BA: Increased Body Awareness
BS_D: Disembodiment
BS_T: Bodily Self-Transcendence
Other_energy: Experiencing the Body as Energy
Other_impermanence: Impermanence of the Body
Othernegative: Negative Body-related Experience
No_BRE: Unidentified
NoTheme: No bodily aspects

BAS_sum.2: The Body Appreciation Scale 2 (BAS-2) score (mean of all items)

ESP_sum2: The Eating Disorder Screen for Primary Care (ESP) score (sum of all items)

sex_dummy: 0 Female; 1 Male.

Non_Class_Psych: Experience facilitated with non-classical psychedelics only 1/0
Class_Psych: Experience facilitated with classical psychedelics only 1/0
Combined_C_NC: Experience facilitated with a combination of classical and non-classical psychedelics 1/0

aff_att_pooled: The reports included one or more of the Affective-Attitudinal Body Image subthemes 1/0
embodi_pooled: The reports included one or more of the Embodiment subthemes 1/0
othernoneg_pooled: The reports included one or more of the Other subthemes 1/0 (Negative Body-related Experience not included)
