# Supplementary material for: Body image phenomenology in reports of psychedelic and meditative experiences: main themes and associations with body attitudes
Source: Neurosci Conscious. 2026 Sep 21;2026(1):niag062. doi: 10.1093/nc/niag062 (PMC13592169; doi:10.1093/nc/niag062)
Supplement: Supplementary file 1 [file supplementary_material_niag062.zip › Supplementary material.pdf]

Supplementary Material 1. Codebook for body-related experiences

General instructions

The contents are rated into themes and subthemes (see below, dichotomic rating of 1 or 0): does a given theme occur or not. Several themes, both main themes and subthemes, may occur within one report.

Within a report, copy-paste the sequence ("sq") of text that represents a specific theme to the respective column (e.g., "PBI_sq"; see below).

If in column "body_1yes_2no_3dontknow" the value is 1 (yes) or 3 (don't know), the rater reads the thematic body report in the column "Thematic_body". If the value is 2 (no), the coding left blank.

If the theme can be identified directly from "Thematic_body", then there's no need to read the longer, open report (column "Open_report").

If the theme is unclear based on the "Thematic_body", or if the participant refers to their open report, then the rater also reads the open report and rates based on that (in addition to the thematic body report).

If the participant reports several experiences, the data is excluded and categorized as missing ("NA:several").

If the fields are left empty, code as missing ("NA:missing").

If there is no body-related experience that can be identified, code as "No identifiable body-related experience".

If the insight came after the acute experience and there is no insight in the acute experience itself, code as "No identifiable body-related experience".

The themes

1. Altered perceptual body image (PBI)
Perceptual body image refers to the mental image or representation of the body based on sensory inputs and visual perception. It involves how we perceive and interpret the size, shape, proportions, and appearance of our body. "Altered perceptual body image" refers to changes and updates in this representation, such as:
• *Perceptual change*, e.g., "My body morphed in shape or size", "I looked different in the mirror", "I felt like I was more beautiful/ugly than usually", "I felt leaner/softer/wrinklier/etc."
• *Updated representation*, e.g., "I realized how big/skinny I had become."

2. Affective-attitudinal body image (ABI)
Affective-attitudinal body image encompasses the feelings and attitudes one holds toward the appearance, functional capacities, or inherent worth of one's body. The subthemes may coincide or occur separately.

2a. Body appreciation (ABI:B)
Body appreciation represents a respectful and accepting attitude that values the body holistically, acknowledging its intrinsic worth while also appreciating its unique features.
• e.g., "I felt loving, caring feelings for my body", "I was able to accept my body", "I found new kinds of beauty in my body."
• "I respect my body and want to live harmoniously with it."
• "The body is a precious gift, and I should treat it well."

2b. Functionality appreciation (ABI:F)

Functionality appreciation describes the recognition of the body's significance as the medium through which life is navigated. The body is valued as a function rather than as an object.

• e.g., "I felt gratitude for the way my body carries me through life and allows me to experience it", "My body is a miracle for all it can do", "It lets me connect with my loved ones and communicate."

• "I stopped worrying about my looks. My body works and that's what matters", "I felt gratitude for my health and my body's ability to heal".

• "I appreciated my body for being a vehicle for my soul."

3. Increased body awareness (BA)

Body awareness refers to the conscious experiencing of bodily sensations, movements and position via an embodied first-person perspective. It includes one's sense of body connection, attunement to bodily signals, and the ability to self-regulate based on these sensations. "Increased body awareness" can be identified by one or more of the following:

• *Sensitivity to internal sensations (interoception)*, e.g. "I became very aware of my bodily processes", "My body felt so alive", "I felt emotions strongly in my body."

• *Sensitivity to sensations of position, pressure, and movement (proprioception)*, e.g., "My body felt heavy and relaxed / energetic and light", "Moving felt effortless and fulfilling. I felt agile, like a dancer."

• *Sense of body connection*, e.g., "I felt at home in my body", "I felt grounded and present in my body", "My body felt comfortable", "I am my body."

• *Self-regulation*, e.g. "I could calm myself with my breath", "I was able to heal old hurt in my body", etc.

• *Overall sense of embodiment*, e.g., "The experience felt embodied."

4. Altered sense of bodily self (BS)

Altered sense of bodily self refers to anomalies in the experience of oneself as an embodied entity. It involves aberrations in body awareness, body ownership (the sensation of one's body as one's own), and self-location (the perceived spatial placement of oneself both within the physical bodyand relative to the surrounding environment). It also encompasses experiences of expanding boundaries and the self being recontextualized into a larger whole. The subthemes may coincide or occur separately.

4a. Disembodiment (BS:D)

Disembodiment refers to a disconnected body experience where one's sense of 1) body awareness, 2) body ownership, and/or 3) self-location is disrupted. This can manifest as:

• *Feelings of not having a body anymore*, e.g., "My body was no longer there. I wasn't aware of having a body at all", "I lost all bodily sensation."

• *Feeling like one's body isn't their own*, e.g., "My body felt alien to me", "I felt like my body was that of someone else."

• *Out-of-body experiences*, e.g., "It seemed as if I was outside my body."

• *Feelings of floating*

• *Feelings of the body dissolving*, e.g., "My body melted", "My body merged with my surroundings / another person."

4b. Bodily self-transcendence (BS:T)

"Bodily self-transcendence" refers to 1) shifts in body boundaries, and 2) the self integrating into a broader context in which it is no longer experienced as being confined within the boundaries of the body. This includes aspects of:

• *Expansiveness*, e.g., "My boundaries expanded and became translucent".

• *Recontextualization*, e.g., "I realized my body is part of a bigger whole", "I stopped identifying with my body, and my 'self' blended with the universal consciousness."

5. Other body-related experience (O)

5a. Experiencing the body as energy (O:E)

• e.g., "I felt like my body was a flux of vibrating movement", "I experienced my body as waves of electricity", "A current / force passed through me."

5b. Impermanence of the body (O:I)

- e.g., “I realized the body is just transitory”, “I understood my body is in constant change.”
- 5c. Negative body-related experience (O:N)
- e.g., “I was disgusted by my body”, “I feared losing control of my body”, “I felt like a prisoner in my body”.

5d. Other body-related experience (O:O)

6. No identifiable body-related experience (N)

Supplementary Material 2. The identified body image themes in detail

1. Altered Perceptual Body Image

Perceiving one’s visual appearance, size or shape in a new or unusual way was present in 9 % of psychedelic narratives. The alterations ranged from featural and detail-oriented (“My hands looked bigger,” female, 22, LSD), to holistic (“It was as if [my body] was ten feet long,” male, 52, LSD). Some implied an affective valence: “[I felt] light, beautiful, smaller, tinier, softer…” (female, 28, psilocybin).

Creative associations to fantastical creatures were described: “I was astonished by my own reflection in the mirror. I looked like a forest troll, though I was still clearly recognizable as myself” (male, 25, psilocybin); “My body felt and looked funny to me, like the body of some humanoid fairytale creature” (female, 27, psilocybin). For some, the perceptions were not only visual, but affected their body schema: “I felt my body had extra parts in it that moved. They were wings, scales or feathers that were attached to my back” (male, 31, psilocybin). One participant also described losing access to their usual appearance: “My face looked different from the mirror, and I didn't remember anymore how I really looked” (female, 22, LSD).

2. Body Appreciation

Appreciative, accepting, and caring attitudes toward one’s body were described in 9 % psychedelic and 2 % of meditation reports. An emotional quality of relief was common to them, as described by a participant during the practice of loving-kindness meditation toward the end of a Vipassana retreat: “The deep appreciation and acceptance, the care I felt, was touching. During the experience, yes, but maybe more importantly after it. All the judging, evaluating, pushing and shaming melt away” (female, 29, metta and vipassana).

Reports with the body appreciation theme commented on physical appearance through holistic self-acceptance rather than self-objectifying evaluation: “I felt like everything got the right proportions. I felt that I'm supposed to be like this” (female, 42, psilocybin). In another example, a participant reported improved appearance satisfaction in the thematic report, “I noticed that my leg muscles were beautifully developed, my skin was soft, and [I] didn't draw attention to those parts of my body that I usually consider less beautiful” (female, 46, MDMA). The open report revealed this to be in the context of appreciating one’s “soul”:

> “First I met my body in its abstract form in the reflection of the candle in the glass of the fireplace stove, and then my soul. I was clearly aware that these were reflections of light, but I was also sure that these were parts of me made by those very reflections. (…) I had the feeling that I was floating in a warm and soft liquid, I felt very comfortable in the physical sense of the word, everything was soft and pleasant, I danced and talked and examined my soul. The body then disappeared but I was overjoyed with my soul.”
> (female, 46, MDMA)

Challenging body-related emotions were not always resolved with increased appearance satisfaction. A male participant reported gaining a more differentiated body attitude during a psilocybin experience, expressing a motivation to lose weight in a healthy way:

> “Initially I felt disgusted. I gained 25 kg in the past 10 years. But later in the trip I realized that the

> solution is simple. I also came to accept my weight in the sense that I don't hate myself for it anymore. Although I still feel the need to lose weight, I came to the insight that it can happen over time, in a healthy way."
> (male, 31, psilocybin)

In some reports, emotional breakthroughs were integral to improved body appreciation. For example, a participant described being able to let go of difficult body-related feelings caused by sexual trauma: "I felt like the experience allowed me to reclaim my body in a way, that some residual guilt and shame I felt around my body disappeared" (male, 30, LSD).

3. Functionality Appreciation

Functionality appreciation, in which the participants articulated appreciation or gratitude distinctly for the body's ability to function and allow one to live, heal, connect, and experience life, was present in 6 % of psychedelic reports and 5 % of meditation reports. Several of the cases expressed gratitude for the body's function as a "vessel": "[I] experienced a sense of appreciation towards my body for being my vessel and carrying me through this life" (female, 28, psilocybin and LSD); "This machine is better than any computer ever created. I have been blessed with it and its shortcomings for me to explore, endure and experience" (male, 39, LSD, psilocybin, and MDMA).

Functionality appreciation coincided with body appreciation in some reports that also valued the body as an object: "[I] understood that it's not ugly, it's just an instrument to live here, to be here and to feel. [I] understood that [I] don't want to be in war with myself for all of my life," (female, 21, LSD); "I understand my body as a gift that provides the framework for allowing my consciousness to be present/embodied in this human world" (female, 64, shamanic sound work). However, some expressed affective indifference toward it outside its functional value: "My bodily perspective was as container and vehicle for this temporary construct of lower identity and mind, therefore essential but not otherwise significant" (male, 72, silent meditation).

4. Increased Body Awareness

Strengthened body connection, attunement to bodily signals, and an overall sense of embodiment were described in 25 % of psychedelic and 15 % of meditation reports: "I remember feeling more at home in my body, feeling much more connecting [sic] to the world in an embodied and tactile way" (female, 39, MDMA). Some participants related these experiences with health and healing:

> "In general during an experience I have felt very strongly the separation of body and soul. In the state you are in during an experience, the border between body and soul become very blurry.Body isn't something you think much about, it's just a vessel, but in this experience I described I felt very much like I was in my body, as I had so much healing done to it."
> (female, 42, ayahuasca)

Embodied experiences were also described in terms of the body being open, co-operative, or very much alive: "I had a sense of the entire body being extraordinarily alive, and that the experience of walking was strongly present and energetic" (female, 41, zazen). A sense of ease and comfort with one's body was felt positively:

> "My body felt as light as a feather and every move I made felt effortless. I also felt really connected to my body and experienced a sense of appreciation towards my body for being my vessel and carrying me through this life. This was somewhat of a significant experience as I was born with a foot deformity and have had to undergo multiple operations to be able to walk. Spending many of my childhood years in casts led to me often being bullied, causing me to develop serious body issues and even a hatred towards my body."
> (female, 28, LSD and psilocybin)

Sharpened interoception was common: "[My] bodily sensations became somehow more sharp and vivid" (male, 28, psilocybin). Some reported learning new ways to self-regulate based on interoceptive awareness: "The sensation of sacred and sensation of effort are extremely subtle and seem to be near throat. These are meaningful because I can remember how they are and have some access to sacred during ... dualistic periods" (other, 41, silent meditation).

5. Disembodiment (Embodiment subtheme)

A variety of intense experiences were reported in which one's bodily self disintegrated, and body awareness, body ownership, or the sense of having an intact body were lost. This was a

predominantly psychedelic theme, found in 27 % of the psychedelic and 13 % of the meditation reports. Disembodiment was described in terms of, e.g., melting, “not having a body at all”, or floating: “My proprioception disappeared completely, and I was a consciousness floating in a void” (male, 46, psilocybin). Some described their body merging with their surroundings or another person (“[I felt] my body evolve to be one with my girlfriend”, male, 25, LSD and MDMA). Although the awareness of the body as a solid, bounded entity was lost, increased sensory sensitivity was reported: “The sense of body disappeared, some moments there were more like, just sensations without the body, merging with the sensations” (female, 29, LSD). This was reported similarly in the meditation reports with the Disembodiment theme:

> “As it ‘melted’ I scanned down into my feet, and I couldn’t distinguish a context, my feet were pure sensation and they felt as if they had dissolved. As I scanned upwards more of the confines of my body dissolved. I went from feeling like I was the one breathing to feeling like I was BEING Breathed. (…) I felt my face disappear and for several minutes I lost the ability to know myself inthe context of my body. After a few minutes I felt myself resolidify from head to toe. Although I felt like I had a different body then [sic] before, with different solidity.”
> (female, 54, vipassana)

6. Bodily Self-Transcendence
Some participants described their body contours becoming transparent and their perception of self expanding beyond their body boundaries, all the while retaining their sense of body connection and presence: “I felt very firmly rooted in my body but also like I was expanding outward into the universe” (female, 41, vipassana). Phenomenologically they were not disembodied, but rather, “transcended” their body and recontextualized their experience of self into a larger whole. This phenomenon was seen predominantly in the meditation reports, with 26 % of these describing it. A significantly smaller occurrence was seen in the psychedelic reports, with 5 % of these including this theme.

Many participants reporting this theme referred to body boundaries and expansion: “The boundary of my skin ceased to be the edge of ‘me’” (male, 27, dzogchen); “My body seemed to expand so vast that it could contain all the universe (…) My body seemed just to be a vessel I am in at the moment, not the true me or in any way limiting my existence” (female, 43, silent meditation). The body was also perceived as a functional part of a greater context:

> “My body was doing things just like it had been always, but I didn't identify with it, it was more like a part of the whole world or environment, just like the road and the air and the trees outside when I was walking.”
> (male, 48, zazen)

7. Body as Energy
Some reports described experiencing their body as an electrical field or a flux of vibrating particles, or they experienced unusual “waves of energy” passing through them. These instances were coded as their own subtheme because of their particularity and recurrence. Present in 18 % of the meditation and 7 % of the psychedelic reports, this was a mostly meditation-related theme. In some cases, the the body was reframed in a new way based on the experience: “I was made aware of the bodies’ vibrational structure” (male, 53, mescaline). This was considered to be healing by the participant, who had suffered physical illness.

This theme co-occurred with disembodiment: “I lost the ability to perceive my body at all, I was just a cloud of energy” (male, 46, vipassana). It also seemed to highlight a sharpened awareness of bodily sensations: “My body was pure sensation – it’s hard to explain, but [it] felt like pulsing energy” (female, 54, vipassana). In some energy-like experiences, high body awareness and the loss of body boundaries coincided, like in this meditation report: “The physical body was here and present, fully awake and aware. During the experience the contours of the body become erased and there is only energy discernable. Inspite (sic) of the felt sense of sitting on the floor” (female, 64, vipassana).

8. Impermanence
In 7 % of the meditation reports, insights into the body’s temporary or constantly changing nature were stated: “[I] woke up to experiencing my entire mind body phenomenon changing on every moment” (male, 64, anapanasati and vipassana). All reporting these insights were vipassana

practitioners. This theme was not present in the psychedelic reports.

9. Negative Body-Related Experience
Negative body-related experiences were described in 3 % of the psychedelic reports. Challenging aspects and difficult emotions would not be considered negative if they were resolved and lead to relief or insight. If the difficulty was too intense to manage, or could not be resolved within the experience, the case would be coded as negative.

Fear and discomfort of losing control over one's bodily processes was common to all cases reporting this theme. In one report, the participant recounted eating a large dose of cannabis, which resulted in intense confusion, fear of dying, and worsening physical pain as the participant tried to stay active for hours with the help of physical exercises. Others reported extreme and unpleasant heaviness, inability to move, and a fear of strange physical sensations. In half of the cases, excessive polydrug use was present. In hindsight, the experiences were considered important by the participants: "It was harsh, but I realised the 3 key problems in my life and then went to a psychiatrist to start psychotherapy. It was a bad moment but necessary to move on and start therapy" (female, 24, LSD, ketamine, and cannabis).

10. Unidentified
In 17 % of the psychedelic, and 15 % of the meditation reports, no body-related themes could be directly identified despite the participant responding positively to the presence of bodily aspects with multiple choice in the thematic question. No interpretations were made beyond explicit statements, although some reports seemed to imply body-related phenomena.

For example, it was concluded that simply reporting "oceanic boundlessness" or "ego dissolution" may or may not imply a bodily experience. They were not coded as disembodiment unless the report explicitly related them to the body. Phrases like "I completely forgot about my body for a while", or "I was spontaneously not attentive to my body" were also not taken to mean disembodiment, but rather, inattentiveness to the body.

Statements such as "feeling relaxed" were not interpreted to mean increased body awareness, even if being relaxed and comfortable could signify positively felt embodiment. The reports had to express that the comfort was exceptional. Moreover, encountering past trauma and emotional breakthroughs were not coded as increased body awareness, although such experiences may have been likely to involve a more direct embodiment of emotion than typical for these participants. While the emotional realm is inherently somatic, the meaning-making and narrative aspects of experiencing emotion were often emphasized more in these narratives. It was decided that unless the participant discussed bodily aspects explicitly, it was not assumed that they were in focus.

11. No bodily aspects
In both psychedelic and meditation samples, 23 % reported that their experience did not involve any personally meaningful body-related aspects.

Supplementary Material 3. Non-significant regression models

Eating disorder traits in relation to the themes in the reports

Nineteen separate binary logistic regression models were performed with each individual theme occurrence (0/1) as the dependent variable and Eating Disorder Screen for Primary Care (ESP) scores, age, and sex (binary) as predictors. The models were performed separately for the psychedelic (N = 116) and meditation (N = 59) reports. The following models were not significant:

Altered Perceptual Body Image in a psychedelic reports, $\chi 2(3) = 3.36$, p = .340. Increased Body Awareness in psychedelic reports, $\chi 2(3) = 1.73$, p = .630. Disembodiment in psychedelic reports, $\chi 2(3) = 0.95$, p = .813. Bodily Self-Transcendence in psychedelic reports, $\chi 2(3, N = 116) = 2.40$, p

= .494. Experiencing the Body as Energy in psychedelic reports, $\chi 2(3) = 3.99$, $p = .263$. Negative Body-Related Experience in psychedelic reports, $\chi 2(3) = 3.75$, $p = .290$. Unidentified in psychedelic reports, $\chi 2(3) = 2.08$, $p = .557$. No bodily aspects in psychedelic reports, $\chi 2(3) = 4.68$, $p = .197$.

Functionality Appreciation in meditation reports, $\chi 2(3) = 2.58$, $p = .462$. Disembodiment in meditation reports, $\chi 2(3) = 2.98$, $p = .405$. Bodily Self-Transcendence in meditation reports, $\chi 2(3) = 3.50$, $p = .320$. Experiencing the Body as Energy in meditation reports, $\chi 2(3) = 3.70$, $p = .295$. Impermanence of the Body in meditation reports, $\chi 2(3) = 6.72$, $p = .081$. Unidentified in meditation reports, $\chi 2(3) = 2.15$, $p = .543$. No bodily aspects in meditation reports, $\chi 2(3) = 1.47$, $p = .689$.
