# Supplementary material for: Body image phenomenology in reports of psychedelic and meditative experiences: main themes and associations with body attitudes
Source: Neurosci Conscious. 2026 Sep 21;2026(1):niag062. doi: 10.1093/nc/niag062 (PMC13592169; doi:10.1093/nc/niag062)
Supplement: Supplementary file 1 [file supplementary_material_niag062.zip › Complete questionnaire - Trip reports - Meditation.docx]

**Study about meditation experiences**

**INTRODUCTION AND INFORMED CONSENT**
Thank you for showing interest in our study! Before deciding whether you want to participate, please read through the following information carefully.

**Contents**
This study is about the contents of meditation experiences. We would like you to choose **one single experience** that you wish to report. This experience should be the one that you personally consider as **the most meaningful or important**. We are interested in all aspects of the experience, and all kinds of experiences, as long as it is facilitated by meditation (e.g. silent meditation, chanting, Holotropic breathing, yoga etc.)

The study contains open questions about the meditation experience where you can tell us about the experience in your own words. Additionally, the study contains questions about your background, such as age, income level, worldview, and possible psychiatric conditions. You will also answer some standardized questionnaires.

**Participation**
- You must be at least 18 years old to participate.
- Please do not respond to the survey more than once.
- You need to complete the study within a single session. The length of the study is approximately 30-60 minutes, depending on how elaborate your answers are.
- Participation is voluntary. You are free to decline your participation at any time without giving a reason. You may discontinue by simply closing the browser window.
- Please note that we will not provide you with personal feedback of any kind and you will not be paid for participating.

**Additional Information**
This study is conducted by researchers at Åbo Akademi University, University of Turku, and Stanford University. The principal investigators are Dr. Jussi Jylkkä (dep. of psychology, Åbo Akademi University, Finland) and Dr. Sikka Pilleriin (dep. of psychology, Stanford University, USA). If you have any questions regarding this study, please contact us at jjylkka@abo.fi. All correspondence is handled confidentially.

You remain completely anonymous to us throughout the study. Data in completely anonymized form will be published according to Open Science principles after the data gathering is finished. We do not gather personal data or IP-address.

**Processing of research data**
Research data from our participants is processed in accordance with the Åbo Akademi University regulations that follow the EU General Data Protection Regulation (GDPR). For more information, please see https://www.abo.fi/en/processing-of-personal-data-at-abo-akademi-university/


**By clicking on the box below with the text “Start experiment”, you confirm that you have read the text above, are 18 years or older, and are willing to take part in this study.**

/title

Background information

/p

/p

Please answer the questions below truthfully. Note that participation is completely voluntary. You can withdraw from the study at any time simply by closing the browser tab or window. <br><br>

/ts + large

1. Age

/p

/p

How old are you (in years)?

/numberfield {

optional: false,

dbcolumn: "age",

numeric: true,

label: "",

value: ,

minimum: 18,

maximum: 150,

increment: 1

}

/lb

/spacer

/p

/ts + large

2. Gender

/singleselect {

optional: false,

default_value: "",

dbcolumn: "gender_1F_2M_3O",

options:

[[

{

dbvalue: "1",

text: "female",

checked: false

},

{

dbvalue: "2",

text: "male",

checked: false

},

{

dbvalue: "3",

text: "other",

checked: false

}

]]

}

/p

/p

/ts + large

3. Education and income

/p

/p

Please choose the highest education level that you have completed:

/singleselect {

optional: false,

default_value: "",

dbcolumn: "education_level",

inline:true,

options: [[

{dbvalue:"1", text:"Primary education"},

{dbvalue:"2", text:"Lower Secondary education"},

{dbvalue:"3", text:"Higher Secondary education (for example, high school diploma)"},

{dbvalue:"4", text:"Vocational education"},

{dbvalue:"6", text:"University: Bachelor’s degree"},

{dbvalue:"7", text:"University: Master’s degree"},

{dbvalue:"8", text:"University: Doctoral degree"},

{dbvalue:"999", text:"Other, please specify"}

]]

}

/p

/textbox {

dbcolumn: "education_level_otherSpecification",

label: "",

linebreak: true,

length: 80,

text: "Other education"

}

/lb

/spacer

/p

Please estimate your personal income compared to the average income in your current country of residence:

/singleselect {

optional: false,

default_value: "",

dbcolumn: "income_level",

inline:true,

options: [[

{dbvalue:"1", text:"Much below average"},

{dbvalue:"2", text:"Below average"},

{dbvalue:"3", text:"Average"},

{dbvalue:"4", text:"Above average"},

{dbvalue:"5", text:"Much above average"},

]]

}

/lb

/spacer

/p

/ts + large

4. Country of birth and language

/p

In which country were you brought up? That is, in which country did you spend most of your childhood? (If you don't wish to disclose this information, you can write "NA")

<br>

/textbox {

dbcolumn: "Country_birth",

label: "",

linebreak: false,

length: 100,

text: "Country of birth",

optional:false

}

/p

/p

#Language History Questionnaire, Li et al. 2014

Please rate your ability in writing English

/singleselect {

optional: false,

default_value: "",

dbcolumn: "English_proficiency",

inline:true,

options: [[

{dbvalue:"1", text:"Native-like"},

{dbvalue:"2", text:"Very good"},

{dbvalue:"3", text:"Good"},

{dbvalue:"4", text:"Functional"},

{dbvalue:"5", text:"Fair"},

{dbvalue:"6", text:"Poor"},

{dbvalue:"7", text:"Very poor"}

]]

}

/lb

/spacer

/p

/ts + large

5. Religion

/p

How would you describe your religion?

/singleselect {

optional: false,

default_value: "",

dbcolumn: "religion",

inline:true,

options: [[

{dbvalue:"1", text:"Agnostic"},

{dbvalue:"2", text:"Atheist"},

{dbvalue:"3", text:"Buddhist"},

{dbvalue:"4", text:"Christian"},

{dbvalue:"5", text:"Hindu"},

{dbvalue:"6", text:"Jewish"},

{dbvalue:"7", text:"Muslim"},

{dbvalue:"8", text:"Non-denominational"},

{dbvalue:"9", text:"Spiritual but not religious"},

{dbvalue:"999", text:"Other, please specify"}

]]

}

/p

/textbox {

dbcolumn: "religion_other_spec",

label: "",

linebreak: true,

length: 80,

text: "Other religion"

}

/p

/p

/ts + large

6. Meditation experience

/p

Do you practice meditation?

/singleselect {

optional: false,

default_value: "",

dbcolumn: "meditation_freq",

inline:true,

options: [[

{dbvalue:"0", text:"No"},

{dbvalue:"1", text:"I have tried once or twice but do not practice regularly"},

{dbvalue:"2", text:"Few times per year"},

{dbvalue:"3", text:"Few times per month"},

{dbvalue:"4", text:"Every week"},

{dbvalue:"5", text:"Daily"},

{dbvalue:"6", text:"Several times a day"},

]]

}

/lb

/spacer

/p

For how long have you practiced meditation?

/p

/singleselect {

optional: false,

default_value: "",

dbcolumn: "meditation_howlong",

inline:true,

options: [[

{dbvalue:"0", text:"I do not meditate"},

{dbvalue:"1", text:"Less than a month"},

{dbvalue:"2", text:"1-6 months"},

{dbvalue:"3", text:"7-12 months"},

{dbvalue:"4", text:"Between 1 and 2 years"},

{dbvalue:"5", text:"Between 2 and 5 years"},

{dbvalue:"6", text:"Over 5 years"},

]]

}

/lb

/spacer

/p

What type of meditation do you practice?

/multiselect {

default_value: "0",

dbcolumn: "bg_medit_type",

colalign:false,

options: [[{dbcolumn:"medit_type_silent", dbvalue: "1", text: "Silent sitting or lying down"},

{dbcolumn:"medit_type_voice", dbvalue: "1", text: "Voicework (e.g. chanting)"},

{dbcolumn:"medit_type_breath", dbvalue: "1", text: "Breathwork (intentional manipulation of breath)"},

{dbcolumn:"medit_type_body", dbvalue: "1", text: "Bodywork (e.g. yoga)"},

{dbcolumn:"medit_type_mindfulness", dbvalue: "1", text: "Mindfulness"}

]]

}

/p

/textbox {

dbcolumn: "bg_medit_type_other",

label: "Other, please specify:",

linebreak: false,

length: 100,

text: "Other meditation type",

optional:

}

/p

/p

/ts + large

7. Experience of psychedelics

/p

Have you ever tried <b>classical psychedelics</b> (e.g., LSD, psilocybin / "magic mushrooms", ayahuasca, DMT, 5-MeO-DMT)?

/singleselect {

optional: false,

default_value: "",

dbcolumn: "classicalPsych_frequency",

inline:true,

options: [[

{dbvalue:"0", text:"Never"},

{dbvalue:"1", text:"Once"},

{dbvalue:"2", text:"Twice"},

{dbvalue:"3", text:"3-5 times"},

{dbvalue:"4", text:"6-10 times"},

{dbvalue:"5", text:"10-50 times"},

{dbvalue:"6", text:"Over 50 times"},

]]

}

/lb

/spacer

/p

Please indicate when was the last time you used <b>classical psychedelics</b> (e.g., LSD, psilocybin / "magic mushrooms", ayahuasca, DMT, 5-MeO-DMT)

/singleselect {

optional: false,

default_value: "",

dbcolumn: "TimeSinceLastClassicalPsych",

inline:true,

options: [[

{dbvalue:"0", text:"Never or over a year ago"},

{dbvalue:"1", text:"9-12 months ago"},

{dbvalue:"2", text:"6-9 months ago"},

{dbvalue:"3", text:"3-6 months ago"},

{dbvalue:"4", text:"1-3 months ago"},

{dbvalue:"5", text:"2-4 weeks ago"},

{dbvalue:"6", text:"One week ago"},

{dbvalue:"7", text:"Less than one week ago"},

]]

}

/lb

/spacer

/p

/ts + large

8. Mental health

/p

Have you ever been diagnosed <b>by a medical professional</b> with any of the following conditions:

/p

/multiselect {

default_value: "0",

dbcolumn: "psychiatric_diagnosis",

colalign:false,

options: [[

{dbcolumn:"Psychiatric_No_disclosure", dbvalue: "1", text: "I don't wish to give this information"},

{dbcolumn:"Psychiatric_severe_depression", dbvalue: "1", text: "Severe depression"},

{dbcolumn:"Psychiatric_bipolar", dbvalue: "1", text: "Bipolar disorder"},

{dbcolumn:"Psychiatric_psychosis", dbvalue: "1", text: "Psychosis or schizophrenia"},

{dbcolumn:"Psychiatric_anxiety", dbvalue: "1", text: "Anxiety disorder"},

{dbcolumn:"Psychiatric_ASD", dbvalue: "1", text: "Autism spectrum disorder / Asperger"},

{dbcolumn:"Psychiatric_OCD", dbvalue: "1", text: "Obsessive-compulsive disorder"},

{dbcolumn:"Psychiatric_ADHD", dbvalue: "1", text: "Attention deficit hyperactivity disorder (ADHD or ADD)"},

]]

}

/p

/textbox {

dbcolumn: "psychiatric_other",

label: "Other diagnosed psychiatric or neurological condition, please specify:",

linebreak: false,

length: 100,

text: "Other condition",

optional:

}

/p

/lb

Do you <b>personally think</b> that you have any psychiatric or neurological condition?

/textbox {

dbcolumn: "psychiatric_selfdiagnosed",

label: "If yes, please specify which one:",

linebreak: false,

length: 100,

text: "Self-diagnosed condition",

optional:

}

/p

/lb

If you use psychoactive substances (e.g., alcohol, nicotine, illicit drugs), please rate how your use of such substances affects you and your close ones:

/singleselect {

optional: false,

default_value: "",

dbcolumn: "substance_use_problem",

inline:true,

options: [[

{dbvalue:"0", text:"Very negatively"},

{dbvalue:"1", text:"Slightly negatively"},

{dbvalue:"2", text:"Neither negatively nor positively"},

{dbvalue:"3", text:"Slightly positively"},

{dbvalue:"4", text:"Very positively"},

{dbvalue:"999", text:"I do not use psychoactive substances"},

]]

}

/lb

/spacer

/p

<b> Eating patterns</b>

/p

Are you satisfied with your eating patterns?

/singleselect {

optional: false,

default_value: "",

dbcolumn: "ESP1_INV",

inline:true,

options: [[

{dbvalue:"1", text:"No"},

{dbvalue:"0", text:"Yes"},

]]

}

/spacer

/p

Do you ever eat in secret?

/singleselect {

optional: false,

default_value: "",

dbcolumn: "ESP2",

inline:true,

options: [[

{dbvalue:"0", text:"No"},

{dbvalue:"1", text:"Yes"},

]]

}

/spacer

/p

Does your weight affect the way you feel about yourself?

/singleselect {

optional: false,

default_value: "",

dbcolumn: "ESP3",

inline:true,

options: [[

{dbvalue:"0", text:"No"},

{dbvalue:"1", text:"Yes"},

]]

}

/spacer

/p

Do you currently suffer with or have you ever suffered in the past with an eating disorder?

/singleselect {

optional: false,

default_value: "",

dbcolumn: "ESP4",

inline:true,

options: [[

{dbvalue:"0", text:"No"},

{dbvalue:"1", text:"Yes"},

]]

}

/spacer

/p Top of Form

/title

**Background of The Experience**

/p

Please think of one meditation experience that you consider as the most meaningful or important in your life, in good or bad. <u>Henceforth, we refer to this experience as <b>"The Experience"</b>, and ask you to evaluate that experience, and only that experience.</u>

/lb

/spacer

/p

<b>1. What type of meditation did you use to facilitate The Experience?</b>

/p

/multiselect {

default_value: "0",

dbcolumn: "exp_medit_type",

colalign:false,

options: [[

{dbcolumn:"exp_medit_silent", dbvalue: "1", text: "Silent sitting or lying down"},

{dbcolumn:"exp_medit_voice", dbvalue: "1", text: "Voicework (e.g. chanting)"},

{dbcolumn:"exp_medit_breath", dbvalue: "1", text: "Breathwork (intentional manipulation of breath)"},

{dbcolumn:"exp_medit_body", dbvalue: "1", text: "Bodywork (e.g. yoga)"},

{dbcolumn:"exp_medit_mindfulness", dbvalue: "1", text: "Mindfulness"}

]]

}

/textbox {

dbcolumn: "exp_other_medit",

label: "Other type, please specify:",

linebreak: false,

length: 100,

text: "Other meditation type",

optional:

}

/p

/lb

Did you follow some specific technique or tradition in the meditation (e.g., vipassana, prayer, zazen)?

/textbox {

dbcolumn: "exp_meditation_technique_specific",

label: "If yes, please indicate what kind of technique:",

linebreak: false,

length: 100,

text: "Meditation detail",

optional:

}

/p

/spacer

/lb

<b>2. When did The Experience happen? </b>

/p

/singleselect {

optional: false,

default_value: "",

dbcolumn: "exp_when",

inline:true,

options: [[

{dbvalue:"0", text:"Less than one week ago"},

{dbvalue:"1", text:"One week ago"},

{dbvalue:"2", text:"2–4 weeks ago"},

{dbvalue:"3", text:"1–3 months ago"},

{dbvalue:"4", text:"3–6 months ago"},

{dbvalue:"5", text:"6–12 months ago"},

{dbvalue:"6", text:"1–2 years ago"},

{dbvalue:"7", text:"2–5 years ago"},

{dbvalue:"8", text:"Over 5 years ago"},

]]

}

/p

/spacer

/lb

<b>3. Where did The Experience happen? </b>

/p

/singleselect {

optional: false,

default_value: "",

dbcolumn: "exp_where",

inline:true,

options: [[

{dbvalue:"0", text:"Home or home-like environment"},

{dbvalue:"1", text:"Friend's home or home-like environment"},

{dbvalue:"2", text:"Unknown person's home or home-like environment"},

{dbvalue:"3", text:"Other private space"},

{dbvalue:"4", text:"Nature"},

{dbvalue:"5", text:"Ceremonial space or retreat"},

{dbvalue:"6", text:"Space designed for therapeutic purpose"},

{dbvalue:"7", text:"Public gathering (e.g., festival)"},

{dbvalue:"8", text:"Other public space"},

]]

}

/p

/spacer

/lb

<b>4. Who was present during The Experience? </b>

/p

/singleselect {

optional: false,

default_value: "",

dbcolumn: "exp_who",

inline:true,

options: [[

{dbvalue:"0", text:"Alone"},

{dbvalue:"1", text:"One other person"},

{dbvalue:"2", text:"Several people, all familiar"},

{dbvalue:"3", text:"Several people, some unfamiliar"},

{dbvalue:"3", text:"Several people, all unfamiliar"},

]]

}

/p

/spacer

/lb

<b>5. Was there a guide, facilitator, or therapist present during The Experience? </b>

/p

/singleselect {

optional: false,

default_value: "",

dbcolumn: "exp_guide",

inline:true,

options: [[

{dbvalue:"0", text:"No"},

{dbvalue:"1", text:"Yes, one"},

{dbvalue:"2", text:"Yes, several"},

]]

}

/p

/spacer

/lb

<b>6. What was the main purpose of The Experience? </b>

/p

/multiselect {

default_value: "0",

dbcolumn: "exp_purpose",

colalign:false,

options: [[

{dbcolumn:"exp_purpose_nospecific", dbvalue: "1", text: "No specific purpose / Cannot say"},

{dbcolumn:"exp_purpose_relax_enjoy", dbvalue: "1", text: "To relax and enjoy"},

{dbcolumn:"exp_purpose_spiritual", dbvalue: "1", text: "Spiritual practice"},

{dbcolumn:"exp_purpose_therapeutic", dbvalue: "1", text: "Therapeutic work"},

{dbcolumn:"exp_purpose_curiosity", dbvalue: "1", text: "Curiosity"},

{dbcolumn:"exp_purpose_social", dbvalue: "1", text: "Social reasons"},

{dbcolumn:"exp_purpose_selfimprovement", dbvalue: "1", text: "Self-improvement"},

{dbcolumn:"exp_purpose_distraction", dbvalue: "1", text: "Distraction from problems or boredom"},

{dbcolumn:"exp_purpose_other", dbvalue: "1", text: "Other specific purpose"},

]]

}

/textbox {

dbcolumn: "exp_other_purpose",

label: "Other purpose, please specify:",

linebreak: false,

length: 100,

text: "Other purpose",

optional:

}

/p

/lb

###############

#OLD:

#/singleselect {

#optional: false,

#default_value: "",

#dbcolumn: "exp_why",

#inline:true,

#options: [[

#{dbvalue:"0", text:"No specific purpose / Cannot say"},

#{dbvalue:"1", text:"To relax and enjoy"},

#{dbvalue:"2", text:"Spiritual practice"},

#{dbvalue:"3", text:"Therapeutic work"},

#{dbvalue:"4", text:"Curiosity"},

#{dbvalue:"5", text:"Social reasons"},

#{dbvalue:"6", text:"Self-improvement"},

#{dbvalue:"7", text:"Distraction from problems or boredom"},

#{dbvalue:"8", text:"Other specific purpose"},

#]]

#}

#/p

#/spacer

#/lb

###########

<b>7. Was The Experience planned? </b>

/p

/singleselect {

optional: false,

default_value: "",

dbcolumn: "exp_planned",

inline:true,

options: [[

{dbvalue:"0", text:"Not at all"},

{dbvalue:"1", text:"Somewhat"},

{dbvalue:"2", text:"Yes"},

]]

}

/p

/spacer

/lb

<b>8. Did you have prior knowledge about what to expect? </b>

/p

/singleselect {

optional: false,

default_value: "",

dbcolumn: "exp_expect",

inline:true,

options: [[

{dbvalue:"0", text:"Not at all"},

{dbvalue:"1", text:"Somewhat"},

{dbvalue:"2", text:"Yes"},

]]

}

/p

/spacer

/lb

<b> 9. Was there any kind of psychoactive substance involved?</b>

/singleselect {

optional: false,

default_value: "",

dbcolumn: "exp_medit_drug",

inline:true,

options: [[

{dbvalue:"1", text:"No"},

{dbvalue:"2", text:"Yes, please specify which one"},

]]

}

/p

/textbox {

dbcolumn: "exp_medit_drugSpecification",

label: "",

linebreak: true,

length: 80,

text: "Which substance?"

}

/p

/spacer

/lb

/p

When you have responded to <u>all</u> the questions, please click

/ts + bold

SUBMIT

/ts - bold

to proceed.

/title

Report of The Experience

/p

/p

/p

/ts + large

What did you experience?

/p

/p

Remember that we are interested in The Experience that was facilitated by meditation and that you consider as the most important or meaningful in your life.

/p

Please write down everything you experienced during The Experience (what happened, where, who was present, thoughts, feelings, images, scenarios) as accurately and in as much detail as possible. Remember that every detail is important.

/p

Do not attempt to make The Experience more structured, organized, logical, or complete than how you remember it. Do not change the description with omissions, additions, conclusions, or embellishments.

/p

In case you would like to provide context to aspects of The Experience (e.g., explain how The Experience relates to your everyday life, etc.), please write those comments in parentheses so that the comments are clearly separated from the description of the actual experience you had.

/p

Please do not give the names of real persons or locations so that your anonymity can be maintained. You can replace the names of persons and places with pseudonyms (fake names).

/p

/p

/textarea {

dbcolumn: "Open_report",

rows: 20,

columns: 50,

label: "<i>Please write your report in English below:</i>",

length:1000000

text:""

}

/spacer

/p

/p

/p

When you are done, please click

/ts + bold

SUBMIT

/ts - bold

to proceed to the next section.

/title

Aspects of The Experience 1/3

/p

/p

Did you perceive <b>your body</b> in a different way or did you have any personally meaningful thoughts, feelings, or insights about your body during The Experience?

<br>

/singleselect {

optional: false,

default_value: "",

dbcolumn: "body_yes_no",

options:

[[

{

dbvalue: "1",

text: "Yes",

checked: false

},

{

dbvalue: "2",

text: "No",

checked: false

},

{

dbvalue: "3",

text: "I don't know",

checked: false

}

]]

}

/p

If yes, please describe these aspects in English below.

/p

Please do not give the names of real persons or locations so that your anonymity can be maintained. You can replace the names of persons and places with pseudonyms (fake names).

/textarea {

dbcolumn: "Thematic_body",

rows: 20,

columns: 50,

label: "",

length:1000000

text:""

}

/spacer

/p

/p

When you are done, please click

/ts + bold

SUBMIT

/ts - bold

to proceed to the next section.

/title

Aspects of The Experience 2/3

/p

/p

Did The Experience involve <b>insights</b> (i.e., feeling that you have accurately and deeply understood something)?

<br>

/singleselect {

optional: false,

default_value: "",

dbcolumn: "insight_yes_no",

options:

[[

{

dbvalue: "1",

text: "Yes",

checked: false

},

{

dbvalue: "2",

text: "No",

checked: false

},

{

dbvalue: "3",

text: "I don't know",

checked: false

}

]]

}

/p

If yes, please describe these aspects in English below.

/p

Please do not give the names of real persons or locations so that your anonymity can be maintained. You can replace the names of persons and places with pseudonyms (fake names).

/textarea {

dbcolumn: "Thematic_insights",

rows: 20,

columns: 50,

label: "",

length:1000000

text:""

}

/spacer

/p

/p

/p

When you are done, please click

/ts + bold

SUBMIT

/ts - bold

to proceed to the next section.

/title

Aspects of The Experience 3/3

/p

/p

Did The Experience involve a sense of <b>connection or alienation</b>?

/singleselect {

optional: false,

default_value: "",

dbcolumn: "connection_or_alienation_yes_no",

options:

[[

{

dbvalue: "1",

text: "Yes",

checked: false

},

{

dbvalue: "2",

text: "No",

checked: false

},

{

dbvalue: "3",

text: "I don't know",

checked: false

}

]]

}

/p

If yes, please describe these aspects in English below.

/p

Please do not give the names of real persons or locations so that your anonymity can be maintained. You can replace the names of persons and places with pseudonyms (fake names).

/textarea {

dbcolumn: "Thematic_connection",

rows: 20,

columns: 50,

label: "",

length:1000000

text:""

}

/spacer

/p

/p

/p

When you are done, please click

/ts + bold

SUBMIT

/ts - bold

to proceed to the next section.

/title

Before and after The Experience

/lb

/p

#Pills questions

/ts + bold

As a result of The Experience, have you noticed any persisting increases or decreases in the following?

/ts - bold

/p

/p

/p

How satisfied you are with your life as a whole

/singleselect {

optional: false,

default_value: "-1",

dbcolumn: "preposts_satisfied",

inline:true,

colalign:false,

options: [[{dbvalue: "1", text: "Decreased a lot"},

{dbvalue: "2", text: "Decreased a bit"},

{dbvalue: "3", text: "Not changed (stayed the same)"},

{dbvalue: "4", text: "Increased a bit"},

{dbvalue: "5", text: "Increased a lot"},

]]

}

/p

/spacer

/lb

How positive you feel in your daily life

/singleselect {

optional: false,

default_value: "-1",

dbcolumn: "preposts_positive",

inline:true,

colalign:false,

options: [[{dbvalue: "1", text: "Decreased a lot"},

{dbvalue: "2", text: "Decreased a bit"},

{dbvalue: "3", text: "Not changed (stayed the same)"},

{dbvalue: "4", text: "Increased a bit"},

{dbvalue: "5", text: "Increased a lot"},

]]

}

/p

/spacer

/lb

How negative you feel in your daily life

/singleselect {

optional: false,

default_value: "-1",

dbcolumn: "preposts_negative",

inline:true,

colalign:false,

options: [[{dbvalue: "1", text: "Decreased a lot"},

{dbvalue: "2", text: "Decreased a bit"},

{dbvalue: "3", text: "Not changed (stayed the same)"},

{dbvalue: "4", text: "Increased a bit"},

{dbvalue: "5", text: "Increased a lot"},

]]

}

/p

/spacer

/lb

The extent to which you feel that your life has meaning and purpose

/singleselect {

optional: false,

default_value: "-1",

dbcolumn: "preposts_meaning",

inline:true,

colalign:false,

options: [[{dbvalue: "1", text: "Decreased a lot"},

{dbvalue: "2", text: "Decreased a bit"},

{dbvalue: "3", text: "Not changed (stayed the same)"},

{dbvalue: "4", text: "Increased a bit"},

{dbvalue: "5", text: "Increased a lot"},

]]

}

/p

/spacer

/lb

The extent to which you feel you have peace and harmony in your mind

/singleselect {

optional: false,

default_value: "-1",

dbcolumn: "preposts_peace_harmony",

inline:true,

colalign:false,

options: [[{dbvalue: "1", text: "Decreased a lot"},

{dbvalue: "2", text: "Decreased a bit"},

{dbvalue: "3", text: "Not changed (stayed the same)"},

{dbvalue: "4", text: "Increased a bit"},

{dbvalue: "5", text: "Increased a lot"},

]]

}

/p

/spacer

/lb

#body questions

#21

Your critical thoughts and feelings about your body

/singleselect {

optional: false,

default_value: "",

dbcolumn: "prepost_bodydissat",

inline:true,

colalign:false,

options: [[{dbvalue: "1", text: "Decreased a lot"},

{dbvalue: "2", text: "Decreased a bit"},

{dbvalue: "3", text: "Not changed (stayed the same)"},

{dbvalue: "4", text: "Increased a bit"},

{dbvalue: "5", text: "Increased a lot"},

]]

}

/p

/spacer

/lb

#22

Your acceptance and appreciation for your body

/singleselect {

optional: false,

default_value: "",

dbcolumn: "prepost_bodyappre",

inline:true,

colalign:false,

options: [[{dbvalue: "1", text: "Decreased a lot"},

{dbvalue: "2", text: "Decreased a bit"},

{dbvalue: "3", text: "Not changed (stayed the same)"},

{dbvalue: "4", text: "Increased a bit"},

{dbvalue: "5", text: "Increased a lot"},

]]

}

/p

/spacer

/lb

#23

Your sense of being "at home" in your body

/singleselect {

optional: false,

default_value: "",

dbcolumn: "prepost_embodiment",

inline:true,

colalign:false,

options: [[{dbvalue: "1", text: "Decreased a lot"},

{dbvalue: "2", text: "Decreased a bit"},

{dbvalue: "3", text: "Not changed (stayed the same)"},

{dbvalue: "4", text: "Increased a bit"},

{dbvalue: "5", text: "Increased a lot"},

]]

}

/p

/spacer

/lb

When you have responded to <u>all</u> the questions, please click

/ts + bold

SUBMIT

/ts - bold

to proceed.

/title

Questionnaires 1/5

/lb

/p

#MEQ-30

/ts + bold

Looking back on the entirety of The Experience, please rate the degree to which you experienced the following phenomena at any time during The Experience. Answer each statement according to the feelings, thoughts, and experiences you had at the time of The Experience.

/ts - bold

/p

/p

/p

1.	Freedom from the limitations of your personal self and feeling a unity or bond with what was felt to be greater than your personal self.

/singleselect {

optional: false,

default_value: "-1",

dbcolumn: "MEQ30_Q1_mystical",

inline:true,

colalign:false,

options: [[{dbvalue: "0", text: "None; not at all"},

{dbvalue: "1", text: "So slight cannot decide"},

{dbvalue: "2", text: "Slight"},

{dbvalue: "3", text: "Moderate"},

{dbvalue: "4", text: "Strong (equivalent in degree to any previous strong experience)"},

{dbvalue: "5", text: "Extreme (more than ever before in my life)"},

]]

}

/p

/spacer

/lb

2.	Experience of pure being and pure awareness (beyond the world of sense impressions)

/singleselect {

optional: false,

default_value: "-1",

dbcolumn: "MEQ30_Q2_mystical",

inline:true,

colalign:false,

options: [[{dbvalue: "0", text: "None; not at all"},

{dbvalue: "1", text: "So slight cannot decide"},

{dbvalue: "2", text: "Slight"},

{dbvalue: "3", text: "Moderate"},

{dbvalue: "4", text: "Strong (equivalent in degree to any previous strong experience)"},

{dbvalue: "5", text: "Extreme (more than ever before in my life)"},

]]

}

/p

/spacer

/lb

3.	Experience of oneness in relation to an “inner world” within.

/singleselect {

optional: false,

default_value: "-1",

dbcolumn: "MEQ30_Q3_mystical",

inline:true,

colalign:false,

options: [[{dbvalue: "0", text: "None; not at all"},

{dbvalue: "1", text: "So slight cannot decide"},

{dbvalue: "2", text: "Slight"},

{dbvalue: "3", text: "Moderate"},

{dbvalue: "4", text: "Strong (equivalent in degree to any previous strong experience)"},

{dbvalue: "5", text: "Extreme (more than ever before in my life)"},

]]

}

/p

/spacer

/lb

4.	Experience of the fusion of your personal self into a larger whole.

/singleselect {

optional: false,

default_value: "-1",

dbcolumn: "MEQ30_Q4_mystical",

inline:true,

colalign:false,

options: [[{dbvalue: "0", text: "None; not at all"},

{dbvalue: "1", text: "So slight cannot decide"},

{dbvalue: "2", text: "Slight"},

{dbvalue: "3", text: "Moderate"},

{dbvalue: "4", text: "Strong (equivalent in degree to any previous strong experience)"},

{dbvalue: "5", text: "Extreme (more than ever before in my life)"},

]]

}

/p

/spacer

/lb

5.	Experience of unity with ultimate reality.

/singleselect {

optional: false,

default_value: "-1",

dbcolumn: "MEQ30_Q5_mystical",

inline:true,

colalign:false,

options: [[{dbvalue: "0", text: "None; not at all"},

{dbvalue: "1", text: "So slight cannot decide"},

{dbvalue: "2", text: "Slight"},

{dbvalue: "3", text: "Moderate"},

{dbvalue: "4", text: "Strong (equivalent in degree to any previous strong experience)"},

{dbvalue: "5", text: "Extreme (more than ever before in my life)"},

]]

}

/p

/spacer

/lb

6.	Feeling that you experienced eternity or infinity.

/singleselect {

optional: false,

default_value: "-1",

dbcolumn: "MEQ30_Q6_mystical",

inline:true,

colalign:false,

options: [[{dbvalue: "0", text: "None; not at all"},

{dbvalue: "1", text: "So slight cannot decide"},

{dbvalue: "2", text: "Slight"},

{dbvalue: "3", text: "Moderate"},

{dbvalue: "4", text: "Strong (equivalent in degree to any previous strong experience)"},

{dbvalue: "5", text: "Extreme (more than ever before in my life)"},

]]

}

/p

/spacer

/lb

7.	Experience of oneness or unity with objects and/or persons perceived in your surroundings.

/singleselect {

optional: false,

default_value: "-1",

dbcolumn: "MEQ30_Q7_mystical",

inline:true,

colalign:false,

options: [[{dbvalue: "0", text: "None; not at all"},

{dbvalue: "1", text: "So slight cannot decide"},

{dbvalue: "2", text: "Slight"},

{dbvalue: "3", text: "Moderate"},

{dbvalue: "4", text: "Strong (equivalent in degree to any previous strong experience)"},

{dbvalue: "5", text: "Extreme (more than ever before in my life)"},

]]

}

/p

/spacer

/lb

8.	Experience of the insight that “all is One”.

/singleselect {

optional: false,

default_value: "-1",

dbcolumn: "MEQ30_Q8_mystical",

inline:true,

colalign:false,

options: [[{dbvalue: "0", text: "None; not at all"},

{dbvalue: "1", text: "So slight cannot decide"},

{dbvalue: "2", text: "Slight"},

{dbvalue: "3", text: "Moderate"},

{dbvalue: "4", text: "Strong (equivalent in degree to any previous strong experience)"},

{dbvalue: "5", text: "Extreme (more than ever before in my life)"},

]]

}

/p

/spacer

/lb

9.	Awareness of the life or living presence in all things.

/singleselect {

optional: false,

default_value: "-1",

dbcolumn: "MEQ30_Q9_mystical",

inline:true,

colalign:false,

options: [[{dbvalue: "0", text: "None; not at all"},

{dbvalue: "1", text: "So slight cannot decide"},

{dbvalue: "2", text: "Slight"},

{dbvalue: "3", text: "Moderate"},

{dbvalue: "4", text: "Strong (equivalent in degree to any previous strong experience)"},

{dbvalue: "5", text: "Extreme (more than ever before in my life)"},

]]

}

/p

/spacer

/lb

10.	Gain of insightful knowledge experienced at an intuitive level.

/singleselect {

optional: false,

default_value: "-1",

dbcolumn: "MEQ30_Q10_mystical",

inline:true,

colalign:false,

options: [[{dbvalue: "0", text: "None; not at all"},

{dbvalue: "1", text: "So slight cannot decide"},

{dbvalue: "2", text: "Slight"},

{dbvalue: "3", text: "Moderate"},

{dbvalue: "4", text: "Strong (equivalent in degree to any previous strong experience)"},

{dbvalue: "5", text: "Extreme (more than ever before in my life)"},

]]

}

/p

/spacer

/lb

11.	Certainty of encounter with ultimate reality (in the sense of being able to “know” and “see” what is really real at some point during your experience.

/singleselect {

optional: false,

default_value: "-1",

dbcolumn: "MEQ30_Q11_mystical",

inline:true,

colalign:false,

options: [[{dbvalue: "0", text: "None; not at all"},

{dbvalue: "1", text: "So slight cannot decide"},

{dbvalue: "2", text: "Slight"},

{dbvalue: "3", text: "Moderate"},

{dbvalue: "4", text: "Strong (equivalent in degree to any previous strong experience)"},

{dbvalue: "5", text: "Extreme (more than ever before in my life)"},

]]

}

/p

/spacer

/lb

12.	You are convinced now, as you look back on your experience, that in it you encountered ultimate reality (i.e., that you “knew” and “saw” what was really real).

/singleselect {

optional: false,

default_value: "-1",

dbcolumn: "MEQ30_Q12_mystical",

inline:true,

colalign:false,

options: [[{dbvalue: "0", text: "None; not at all"},

{dbvalue: "1", text: "So slight cannot decide"},

{dbvalue: "2", text: "Slight"},

{dbvalue: "3", text: "Moderate"},

{dbvalue: "4", text: "Strong (equivalent in degree to any previous strong experience)"},

{dbvalue: "5", text: "Extreme (more than ever before in my life)"},

]]

}

/p

/spacer

/lb

13.	Sense of being at a spiritual height.

/singleselect {

optional: false,

default_value: "-1",

dbcolumn: "MEQ30_Q13_mystical",

inline:true,

colalign:false,

options: [[{dbvalue: "0", text: "None; not at all"},

{dbvalue: "1", text: "So slight cannot decide"},

{dbvalue: "2", text: "Slight"},

{dbvalue: "3", text: "Moderate"},

{dbvalue: "4", text: "Strong (equivalent in degree to any previous strong experience)"},

{dbvalue: "5", text: "Extreme (more than ever before in my life)"},

]]

}

/p

/spacer

/lb

14.	Sense of reverence.

/singleselect {

optional: false,

default_value: "-1",

dbcolumn: "MEQ30_Q14_mystical",

inline:true,

colalign:false,

options: [[{dbvalue: "0", text: "None; not at all"},

{dbvalue: "1", text: "So slight cannot decide"},

{dbvalue: "2", text: "Slight"},

{dbvalue: "3", text: "Moderate"},

{dbvalue: "4", text: "Strong (equivalent in degree to any previous strong experience)"},

{dbvalue: "5", text: "Extreme (more than ever before in my life)"},

]]

}

/p

/spacer

/lb

15.	Feeling that you experienced something profoundly sacred and holy.

/singleselect {

optional: false,

default_value: "-1",

dbcolumn: "MEQ30_Q15_mystical",

inline:true,

colalign:false,

options: [[{dbvalue: "0", text: "None; not at all"},

{dbvalue: "1", text: "So slight cannot decide"},

{dbvalue: "2", text: "Slight"},

{dbvalue: "3", text: "Moderate"},

{dbvalue: "4", text: "Strong (equivalent in degree to any previous strong experience)"},

{dbvalue: "5", text: "Extreme (more than ever before in my life)"},

]]

}

/p

/spacer

/lb

16.	Experience of amazement.

/singleselect {

optional: false,

default_value: "-1",

dbcolumn: "MEQ30_Q16_positive_mood",

inline:true,

colalign:false,

options: [[{dbvalue: "0", text: "None; not at all"},

{dbvalue: "1", text: "So slight cannot decide"},

{dbvalue: "2", text: "Slight"},

{dbvalue: "3", text: "Moderate"},

{dbvalue: "4", text: "Strong (equivalent in degree to any previous strong experience)"},

{dbvalue: "5", text: "Extreme (more than ever before in my life)"},

]]

}

/p

/spacer

/lb

17.	Feelings of tenderness and gentleness.

/singleselect {

optional: false,

default_value: "-1",

dbcolumn: "MEQ30_Q17_positive_mood",

inline:true,

colalign:false,

options: [[{dbvalue: "0", text: "None; not at all"},

{dbvalue: "1", text: "So slight cannot decide"},

{dbvalue: "2", text: "Slight"},

{dbvalue: "3", text: "Moderate"},

{dbvalue: "4", text: "Strong (equivalent in degree to any previous strong experience)"},

{dbvalue: "5", text: "Extreme (more than ever before in my life)"},

]]

}

/p

/spacer

/lb

18.	Feelings of peace and tranquility.

/singleselect {

optional: false,

default_value: "-1",

dbcolumn: "MEQ30_Q18_positive_mood",

inline:true,

colalign:false,

options: [[{dbvalue: "0", text: "None; not at all"},

{dbvalue: "1", text: "So slight cannot decide"},

{dbvalue: "2", text: "Slight"},

{dbvalue: "3", text: "Moderate"},

{dbvalue: "4", text: "Strong (equivalent in degree to any previous strong experience)"},

{dbvalue: "5", text: "Extreme (more than ever before in my life)"},

]]

}

/p

/spacer

/lb

19.	Experience of ecstasy.

/singleselect {

optional: false,

default_value: "-1",

dbcolumn: "MEQ30_Q19_positive_mood",

inline:true,

colalign:false,

options: [[{dbvalue: "0", text: "None; not at all"},

{dbvalue: "1", text: "So slight cannot decide"},

{dbvalue: "2", text: "Slight"},

{dbvalue: "3", text: "Moderate"},

{dbvalue: "4", text: "Strong (equivalent in degree to any previous strong experience)"},

{dbvalue: "5", text: "Extreme (more than ever before in my life)"},

]]

}

/p

/spacer

/lb

20.	Sense of awe or awesomeness.

/singleselect {

optional: false,

default_value: "-1",

dbcolumn: "MEQ30_Q20_positive_mood",

inline:true,

colalign:false,

options: [[{dbvalue: "0", text: "None; not at all"},

{dbvalue: "1", text: "So slight cannot decide"},

{dbvalue: "2", text: "Slight"},

{dbvalue: "3", text: "Moderate"},

{dbvalue: "4", text: "Strong (equivalent in degree to any previous strong experience)"},

{dbvalue: "5", text: "Extreme (more than ever before in my life)"},

]]

}

/p

/spacer

/lb

21.	Feelings of joy.

/singleselect {

optional: false,

default_value: "-1",

dbcolumn: "MEQ30_Q21_positive_mood",

inline:true,

colalign:false,

options: [[{dbvalue: "0", text: "None; not at all"},

{dbvalue: "1", text: "So slight cannot decide"},

{dbvalue: "2", text: "Slight"},

{dbvalue: "3", text: "Moderate"},

{dbvalue: "4", text: "Strong (equivalent in degree to any previous strong experience)"},

{dbvalue: "5", text: "Extreme (more than ever before in my life)"},

]]

}

/p

/spacer

/lb

22.	Loss of your usual sense of time.

/singleselect {

optional: false,

default_value: "-1",

dbcolumn: "MEQ30_Q22_transcendence",

inline:true,

colalign:false,

options: [[{dbvalue: "0", text: "None; not at all"},

{dbvalue: "1", text: "So slight cannot decide"},

{dbvalue: "2", text: "Slight"},

{dbvalue: "3", text: "Moderate"},

{dbvalue: "4", text: "Strong (equivalent in degree to any previous strong experience)"},

{dbvalue: "5", text: "Extreme (more than ever before in my life)"},

]]

}

/p

/spacer

/lb

23.	Loss of your usual sense of space.

/singleselect {

optional: false,

default_value: "-1",

dbcolumn: "MEQ30_Q23_transcendence",

inline:true,

colalign:false,

options: [[{dbvalue: "0", text: "None; not at all"},

{dbvalue: "1", text: "So slight cannot decide"},

{dbvalue: "2", text: "Slight"},

{dbvalue: "3", text: "Moderate"},

{dbvalue: "4", text: "Strong (equivalent in degree to any previous strong experience)"},

{dbvalue: "5", text: "Extreme (more than ever before in my life)"},

]]

}

/p

/spacer

/lb

24.	Loss of usual awareness of where you were.

/singleselect {

optional: false,

default_value: "-1",

dbcolumn: "MEQ30_Q24_transcendence",

inline:true,

colalign:false,

options: [[{dbvalue: "0", text: "None; not at all"},

{dbvalue: "1", text: "So slight cannot decide"},

{dbvalue: "2", text: "Slight"},

{dbvalue: "3", text: "Moderate"},

{dbvalue: "4", text: "Strong (equivalent in degree to any previous strong experience)"},

{dbvalue: "5", text: "Extreme (more than ever before in my life)"},

]]

}

/p

/spacer

/lb

25.	Sense of being “outside of” time, beyond past and future.

/singleselect {

optional: false,

default_value: "-1",

dbcolumn: "MEQ30_Q25_transcendence",

inline:true,

colalign:false,

options: [[{dbvalue: "0", text: "None; not at all"},

{dbvalue: "1", text: "So slight cannot decide"},

{dbvalue: "2", text: "Slight"},

{dbvalue: "3", text: "Moderate"},

{dbvalue: "4", text: "Strong (equivalent in degree to any previous strong experience)"},

{dbvalue: "5", text: "Extreme (more than ever before in my life)"},

]]

}

/p

/spacer

/lb

26.	Being in a realm with no space boundaries.

/singleselect {

optional: false,

default_value: "-1",

dbcolumn: "MEQ30_Q26_transcendence",

inline:true,

colalign:false,

options: [[{dbvalue: "0", text: "None; not at all"},

{dbvalue: "1", text: "So slight cannot decide"},

{dbvalue: "2", text: "Slight"},

{dbvalue: "3", text: "Moderate"},

{dbvalue: "4", text: "Strong (equivalent in degree to any previous strong experience)"},

{dbvalue: "5", text: "Extreme (more than ever before in my life)"},

]]

}

/p

/spacer

/lb

27.	Experience of timelessness.

/singleselect {

optional: false,

default_value: "-1",

dbcolumn: "MEQ30_Q27_transcendence",

inline:true,

colalign:false,

options: [[{dbvalue: "0", text: "None; not at all"},

{dbvalue: "1", text: "So slight cannot decide"},

{dbvalue: "2", text: "Slight"},

{dbvalue: "3", text: "Moderate"},

{dbvalue: "4", text: "Strong (equivalent in degree to any previous strong experience)"},

{dbvalue: "5", text: "Extreme (more than ever before in my life)"},

]]

}

/p

/spacer

/lb

28.	Sense that the experience cannot be described adequately in words.

/singleselect {

optional: false,

default_value: "-1",

dbcolumn: "MEQ30_Q28_ineffability",

inline:true,

colalign:false,

options: [[{dbvalue: "0", text: "None; not at all"},

{dbvalue: "1", text: "So slight cannot decide"},

{dbvalue: "2", text: "Slight"},

{dbvalue: "3", text: "Moderate"},

{dbvalue: "4", text: "Strong (equivalent in degree to any previous strong experience)"},

{dbvalue: "5", text: "Extreme (more than ever before in my life)"},

]]

}

/p

/spacer

/lb

29.	Feeling that you could not do justice to your experience by describing it in words.

/singleselect {

optional: false,

default_value: "-1",

dbcolumn: "MEQ30_Q29_ineffability",

inline:true,

colalign:false,

options: [[{dbvalue: "0", text: "None; not at all"},

{dbvalue: "1", text: "So slight cannot decide"},

{dbvalue: "2", text: "Slight"},

{dbvalue: "3", text: "Moderate"},

{dbvalue: "4", text: "Strong (equivalent in degree to any previous strong experience)"},

{dbvalue: "5", text: "Extreme (more than ever before in my life)"},

]]

}

/p

/spacer

/lb

30.	Feeling that it would be difficult to communicate your own experience to others who have not had similar experiences.

/singleselect {

optional: false,

default_value: "-1",

dbcolumn: "MEQ30_Q30_ineffability",

inline:true,

colalign:false,

options: [[{dbvalue: "0", text: "None; not at all"},

{dbvalue: "1", text: "So slight cannot decide"},

{dbvalue: "2", text: "Slight"},

{dbvalue: "3", text: "Moderate"},

{dbvalue: "4", text: "Strong (equivalent in degree to any previous strong experience)"},

{dbvalue: "5", text: "Extreme (more than ever before in my life)"},

]]

}

/p

/spacer

/lb

/title

Questionnaires

/lb

/p

In the following final section of the survey, you will be presented with five brief questionnaires. <b>Please note that the following questionnaires are no longer about The Experience.</b> Please click on "Submit" to continue.

/p

/p

/title

Questionnaires 1/5

/lb

/p

#Short WEMWBS

/ts + bold

Below are some statements about feelings and thoughts.

Please tick the box that best describes your experience of each over the last 2 weeks.

/ts - bold

/p

/p

/p

I’ve been feeling optimistic about the future.

/singleselect {

optional: false,

default_value: "-1",

dbcolumn: "WEMWBS_optimistic",

inline:true,

colalign:false,

options: [[{dbvalue: "1", text: "None of the time"},

{dbvalue: "2", text: "Rarely"},

{dbvalue: "3", text: "Some of the time"},

{dbvalue: "4", text: "Often"},

{dbvalue: "5", text: "All of the time"},

]]

}

/p

/spacer

/lb

I’ve been feeling useful.

/singleselect {

optional: false,

default_value: "-1",

dbcolumn: "WEMWBS_useful",

inline:true,

colalign:false,

options: [[{dbvalue: "1", text: "None of the time"},

{dbvalue: "2", text: "Rarely"},

{dbvalue: "3", text: "Some of the time"},

{dbvalue: "4", text: "Often"},

{dbvalue: "5", text: "All of the time"},

]]

}

/p

/spacer

/lb

I’ve been feeling relaxed.

/singleselect {

optional: false,

default_value: "-1",

dbcolumn: "WEMWBS_relaxed",

inline:true,

colalign:false,

options: [[{dbvalue: "1", text: "None of the time"},

{dbvalue: "2", text: "Rarely"},

{dbvalue: "3", text: "Some of the time"},

{dbvalue: "4", text: "Often"},

{dbvalue: "5", text: "All of the time"},

]]

}

/p

/spacer

/lb

I’ve been dealing with problems well.

/singleselect {

optional: false,

default_value: "-1",

dbcolumn: "WEMWBS_dealing_w_problems",

inline:true,

colalign:false,

options: [[{dbvalue: "1", text: "None of the time"},

{dbvalue: "2", text: "Rarely"},

{dbvalue: "3", text: "Some of the time"},

{dbvalue: "4", text: "Often"},

{dbvalue: "5", text: "All of the time"},

]]

}

/p

/spacer

/lb

I’ve been thinking clearly.

/singleselect {

optional: false,

default_value: "-1",

dbcolumn: "WEMWBS_thinking_clearly",

inline:true,

colalign:false,

options: [[{dbvalue: "1", text: "None of the time"},

{dbvalue: "2", text: "Rarely"},

{dbvalue: "3", text: "Some of the time"},

{dbvalue: "4", text: "Often"},

{dbvalue: "5", text: "All of the time"},

]]

}

/p

/spacer

/lb

I’ve been feeling close to other people.

/singleselect {

optional: false,

default_value: "-1",

dbcolumn: "WEMWBS_close_to_others",

inline:true,

colalign:false,

options: [[{dbvalue: "1", text: "None of the time"},

{dbvalue: "2", text: "Rarely"},

{dbvalue: "3", text: "Some of the time"},

{dbvalue: "4", text: "Often"},

{dbvalue: "5", text: "All of the time"},

]]

}

/p

/spacer

/lb

I’ve been able to make up my own mind about things.

/singleselect {

optional: false,

default_value: "-1",

dbcolumn: "WEMWBS_make_up_my_own_mind",

inline:true,

colalign:false,

options: [[{dbvalue: "1", text: "None of the time"},

{dbvalue: "2", text: "Rarely"},

{dbvalue: "3", text: "Some of the time"},

{dbvalue: "4", text: "Often"},

{dbvalue: "5", text: "All of the time"},

]]

}

/p

/spacer

/lb

/p

When you have responded to <u>all</u> the questions, please click

/ts + bold

SUBMIT

/ts - bold

to proceed.

/title

Questionnaires 2/5

/lb

/p

#Peace of Mind Scale (PoMS), 7 items

/ts

<b>How often do you feel internal peace and ease in your daily life? Use the following scale to indicate your response.</b>

/ts

/p

/p

/lb

My mind is free and at ease.

/singleselect {

optional: false,

default_value: "-1",

dbcolumn: "PoMS_1",

inline:true,

colalign:false,

options: [[{dbvalue: "1", text: "Not at all"},

{dbvalue: "2", text: "Some of the time"},

{dbvalue: "3", text: "Often"},

{dbvalue: "4", text: "Most of the time"},

{dbvalue: "5", text: "All of the time"},

]]

}

/p

/spacer

/lb

I feel content and comfortable with myself in daily life.

/singleselect {

optional: false,

default_value: "-1",

dbcolumn: "PoMS_2",

inline:true,

colalign:false,

options: [[{dbvalue: "1", text: "Not at all"},

{dbvalue: "2", text: "Some of the time"},

{dbvalue: "3", text: "Often"},

{dbvalue: "4", text: "Most of the time"},

{dbvalue: "5", text: "All of the time"},

]]

}

/p

/spacer

/lb

My lifestyle gives me feelings of peace and stability.

/singleselect {

optional: false,

default_value: "-1",

dbcolumn: "PoMS_3",

inline:true,

colalign:false,

options: [[{dbvalue: "1", text: "Not at all"},

{dbvalue: "2", text: "Some of the time"},

{dbvalue: "3", text: "Often"},

{dbvalue: "4", text: "Most of the time"},

{dbvalue: "5", text: "All of the time"},

]]

}

/p

/spacer

/lb

I have peace and harmony in my mind.

/singleselect {

optional: false,

default_value: "-1",

dbcolumn: "PoMS_4",

inline:true,

colalign:false,

options: [[{dbvalue: "1", text: "Not at all"},

{dbvalue: "2", text: "Some of the time"},

{dbvalue: "3", text: "Often"},

{dbvalue: "4", text: "Most of the time"},

{dbvalue: "5", text: "All of the time"},

]]

}

/p

/spacer

/lb

It is difficult for me to feel settled.

/singleselect {

optional: false,

default_value: "-1",

dbcolumn: "PoMS_5_INV",

inline:true,

colalign:false,

options: [[{dbvalue: "1", text: "Not at all"},

{dbvalue: "2", text: "Some of the time"},

{dbvalue: "3", text: "Often"},

{dbvalue: "4", text: "Most of the time"},

{dbvalue: "5", text: "All of the time"},

]]

}

/p

/spacer

/lb

The way I live brings me feelings of peace and comfort.

/singleselect {

optional: false,

default_value: "-1",

dbcolumn: "PoMS_6",

inline:true,

colalign:false,

options: [[{dbvalue: "1", text: "Not at all"},

{dbvalue: "2", text: "Some of the time"},

{dbvalue: "3", text: "Often"},

{dbvalue: "4", text: "Most of the time"},

{dbvalue: "5", text: "All of the time"},

]]

}

/p

/spacer

/lb

I feel anxious and uneasy in my mind.

/singleselect {

optional: false,

default_value: "-1",

dbcolumn: "PoMS_7_INV",

inline:true,

colalign:false,

options: [[{dbvalue: "1", text: "Not at all"},

{dbvalue: "2", text: "Some of the time"},

{dbvalue: "3", text: "Often"},

{dbvalue: "4", text: "Most of the time"},

{dbvalue: "5", text: "All of the time"},

]]

}

/p

/spacer

/lb

/p

When you have responded to <u>all</u> the questions, please click

/ts + bold

SUBMIT

/ts - bold

to proceed.

/title

Questionnaires 3/5

/lb

/p

#The Body Appreciation Scale-2

/p

/ts + bold

The next statements are about your relationship to your body. Please indicate whether the question is true about you never, seldom, sometimes, often, or always.

/ts - bold

/p

/lb

#1

I respect my body.

/singleselect {

optional: false,

default_value: "-1",

dbcolumn: "BAS1",

inline:true,

colalign:false,

options: [[{dbvalue: "1", text: "Never"},

{dbvalue: "2", text: "Seldom"},

{dbvalue: "3", text: "Sometimes"},

{dbvalue: "4", text: "Often"},

{dbvalue: "5", text: "Always"},

]]

}

/p

/spacer

/lb

#2

I feel good about my body.

/singleselect {

optional: false,

default_value: "-1",

dbcolumn: "BAS2",

inline:true,

colalign:false,

options: [[{dbvalue: "1", text: "Never"},

{dbvalue: "2", text: "Seldom"},

{dbvalue: "3", text: "Sometimes"},

{dbvalue: "4", text: "Often"},

{dbvalue: "5", text: "Always"},

]]

}

/p

/spacer

/lb

#3

I feel that my body has at least some good qualities.

/singleselect {

optional: false,

default_value: "-1",

dbcolumn: "BAS3",

inline:true,

colalign:false,

options: [[{dbvalue: "1", text: "Never"},

{dbvalue: "2", text: "Seldom"},

{dbvalue: "3", text: "Sometimes"},

{dbvalue: "4", text: "Often"},

{dbvalue: "5", text: "Always"},

]]

}

/p

/spacer

/lb

#4

I take a positive attitude towards my body.

/singleselect {

optional: false,

default_value: "-1",

dbcolumn: "BAS4",

inline:true,

colalign:false,

options: [[{dbvalue: "1", text: "Never"},

{dbvalue: "2", text: "Seldom"},

{dbvalue: "3", text: "Sometimes"},

{dbvalue: "4", text: "Often"},

{dbvalue: "5", text: "Always"},

]]

}

/p

/spacer

/lb

#5

I am attentive to my body's needs.

/singleselect {

optional: false,

default_value: "-1",

dbcolumn: "BAS5",

inline:true,

colalign:false,

options: [[{dbvalue: "1", text: "Never"},

{dbvalue: "2", text: "Seldom"},

{dbvalue: "3", text: "Sometimes"},

{dbvalue: "4", text: "Often"},

{dbvalue: "5", text: "Always"},

]]

}

/p

/spacer

/lb

#6

I feel love for my body.

/singleselect {

optional: false,

default_value: "-1",

dbcolumn: "BAS6",

inline:true,

colalign:false,

options: [[{dbvalue: "1", text: "Never"},

{dbvalue: "2", text: "Seldom"},

{dbvalue: "3", text: "Sometimes"},

{dbvalue: "4", text: "Often"},

{dbvalue: "5", text: "Always"},

]]

}

/p

/spacer

/lb

#7

I appreciate the different and unique characteristics of my body.

/singleselect {

optional: false,

default_value: "-1",

dbcolumn: "BAS7",

inline:true,

colalign:false,

options: [[{dbvalue: "1", text: "Never"},

{dbvalue: "2", text: "Seldom"},

{dbvalue: "3", text: "Sometimes"},

{dbvalue: "4", text: "Often"},

{dbvalue: "5", text: "Always"},

]]

}

/p

/spacer

/lb

#8

My behavior reveals my positive attitude toward my body; for example, I hold my head high and smile.

/singleselect {

optional: false,

default_value: "-1",

dbcolumn: "BAS8",

inline:true,

colalign:false,

options: [[{dbvalue: "1", text: "Never"},

{dbvalue: "2", text: "Seldom"},

{dbvalue: "3", text: "Sometimes"},

{dbvalue: "4", text: "Often"},

{dbvalue: "5", text: "Always"},

]]

}

/p

/spacer

/lb

#9

I am comfortable in my body.

/singleselect {

optional: false,

default_value: "-1",

dbcolumn: "BAS9",

inline:true,

colalign:false,

options: [[{dbvalue: "1", text: "Never"},

{dbvalue: "2", text: "Seldom"},

{dbvalue: "3", text: "Sometimes"},

{dbvalue: "4", text: "Often"},

{dbvalue: "5", text: "Always"},

]]

}

/p

/spacer

/lb

#10

I feel like I am beautiful even if I am different from media images of attractive people (e.g. models, actresses/actors).

/singleselect {

optional: false,

default_value: "-1",

dbcolumn: "BAS10",

inline:true,

colalign:false,

options: [[{dbvalue: "1", text: "Never"},

{dbvalue: "2", text: "Seldom"},

{dbvalue: "3", text: "Sometimes"},

{dbvalue: "4", text: "Often"},

{dbvalue: "5", text: "Always"},

]]

}

/p

/spacer

/lb

/p

When you have responded to <u>all</u> the questions, please click

/ts + bold

SUBMIT

/ts - bold

to proceed.

/title

Questionnaires 4/5

/lb

/p

#Psy-Flex

/p

/ts + bold

Below are statements about your current psychological flexibility. The questions refer to your experiences in the last seven days.

/ts - bold

/p

/lb

#1

1. Being present.

/p

/p

Even if I am somewhere else with my thoughts, I can focus on what's going on in important moments.

/singleselect {

optional: false,

default_value: "-1",

dbcolumn: "psy_flex1",

inline:true,

colalign:false,

options: [[{dbvalue: "5", text: "Very often"},

{dbvalue: "4", text: "Often"},

{dbvalue: "3", text: "From time to time"},

{dbvalue: "2", text: "Seldom"},

{dbvalue: "1", text: "Very seldom"},

]]

}

/p

/spacer

/lb

#2

2. Being open for experiences.

/p

/p

If need be, I can let unpleasant thoughts and experiences happen without having to get rid of them immediately.

/singleselect {

optional: false,

default_value: "-1",

dbcolumn: "psy_flex2",

inline:true,

colalign:false,

options: [[{dbvalue: "5", text: "Very often"},

{dbvalue: "4", text: "Often"},

{dbvalue: "3", text: "From time to time"},

{dbvalue: "2", text: "Seldom"},

{dbvalue: "1", text: "Very seldom"},

]]

}

/p

/spacer

/lb

#3

3. Leaving thoughts be.

/p

/p

I can look at hindering thoughts from a distance without letting them control me.

/singleselect {

optional: false,

default_value: "-1",

dbcolumn: "psy_flex3",

inline:true,

colalign:false,

options: [[{dbvalue: "5", text: "Very often"},

{dbvalue: "4", text: "Often"},

{dbvalue: "3", text: "From time to time"},

{dbvalue: "2", text: "Seldom"},

{dbvalue: "1", text: "Very seldom"},

]]

}

/p

/spacer

/lb

#4

4. Steady self.

/p

/p

Even if thoughts and experiences are confusing me I can notice something like a steady core inside of me.

/singleselect {

optional: false,

default_value: "-1",

dbcolumn: "psy_flex4",

inline:true,

colalign:false,

options: [[{dbvalue: "5", text: "Very often"},

{dbvalue: "4", text: "Often"},

{dbvalue: "3", text: "From time to time"},

{dbvalue: "2", text: "Seldom"},

{dbvalue: "1", text: "Very seldom"},

]]

}

/p

/spacer

/lb

#5

5. Awareness of one's own values.

/p

/p

I determine what's important for me and decide what I want to use my energy for.

/singleselect {

optional: false,

default_value: "-1",

dbcolumn: "psy_flex5",

inline:true,

colalign:false,

options: [[{dbvalue: "5", text: "Very often"},

{dbvalue: "4", text: "Often"},

{dbvalue: "3", text: "From time to time"},

{dbvalue: "2", text: "Seldom"},

{dbvalue: "1", text: "Very seldom"},

]]

}

/p

/spacer

/lb

#6

6. Being engaged.

/p

/p

I engage thoroughly in things that are important, useful, or meaningful to me.

/singleselect {

optional: false,

default_value: "-1",

dbcolumn: "psy_flex6",

inline:true,

colalign:false,

options: [[{dbvalue: "5", text: "Very often"},

{dbvalue: "4", text: "Often"},

{dbvalue: "3", text: "From time to time"},

{dbvalue: "2", text: "Seldom"},

{dbvalue: "1", text: "Very seldom"},

]]

}

/p

/spacer

/lb

/p

When you have responded to <u>all</u> the questions, please click

/ts + bold

SUBMIT

/ts - bold

to proceed.

/title

Questionnaires 5/5

/lb

/p

#Values

/p

/ts + bold

Please, rate the importance of the following values as a life-guiding principle for you. Use the 8-point scale in which 0 indicates that the value is opposed to your principles, 1 indicates that the values is not important for you, 4 indicates that the values is important, and 8 indicates that the value is of supreme importance for you.

/ts - bold

/p

/lb

#1

1. POWER (social power, authority, wealth)

/singleselect {

optional: false,

default_value: "-1",

dbcolumn: "SVS_1_Power",

inline:true,

colalign:false,

options: [[{dbvalue: "8", text: "8 (Of supreme importance)"},

{dbvalue: "7", text: "7"},

{dbvalue: "6", text: "6"},

{dbvalue: "5", text: "5"},

{dbvalue: "4", text: "4 (Important)"},

{dbvalue: "3", text: "3"},

{dbvalue: "2", text: "2"},

{dbvalue: "1", text: "1 (Not important)"},

{dbvalue: "0", text: "0 (Opposed to my principles)"},

]]

}

/p

/spacer

/lb

#2

2. ACHIEVEMENT (success, capability, ambition, influence on people and	events)

/singleselect {

optional: false,

default_value: "-1",

dbcolumn: "SVS_1_Power",

inline:true,

colalign:false,

options: [[{dbvalue: "8", text: "8 (Of supreme importance)"},

{dbvalue: "7", text: "7"},

{dbvalue: "6", text: "6"},

{dbvalue: "5", text: "5"},

{dbvalue: "4", text: "4 (Important)"},

{dbvalue: "3", text: "3"},

{dbvalue: "2", text: "2"},

{dbvalue: "1", text: "1 (Not important)"},

{dbvalue: "0", text: "0 (Opposed to my principles)"},

]]

}

/p

/spacer

/lb

#3

3. HEDONISM (gratification of desires, enjoyment in life, self-indulgence)

/singleselect {

optional: false,

default_value: "-1",

dbcolumn: "SVS_1_Power",

inline:true,

colalign:false,

options: [[{dbvalue: "8", text: "8 (Of supreme importance)"},

{dbvalue: "7", text: "7"},

{dbvalue: "6", text: "6"},

{dbvalue: "5", text: "5"},

{dbvalue: "4", text: "4 (Important)"},

{dbvalue: "3", text: "3"},

{dbvalue: "2", text: "2"},

{dbvalue: "1", text: "1 (Not important)"},

{dbvalue: "0", text: "0 (Opposed to my principles)"},

]]

}

/p

/spacer

/lb

#4

4. STIMULATION (daring, a varied and challenging life, an exciting life)

/singleselect {

optional: false,

default_value: "-1",

dbcolumn: "SVS_1_Power",

inline:true,

colalign:false,

options: [[{dbvalue: "8", text: "8 (Of supreme importance)"},

{dbvalue: "7", text: "7"},

{dbvalue: "6", text: "6"},

{dbvalue: "5", text: "5"},

{dbvalue: "4", text: "4 (Important)"},

{dbvalue: "3", text: "3"},

{dbvalue: "2", text: "2"},

{dbvalue: "1", text: "1 (Not important)"},

{dbvalue: "0", text: "0 (Opposed to my principles)"},

]]

}

/p

/spacer

/lb

#5

5. SELF-DIRECTION (creativity, freedom, curiosity, independence, choosing one's own goals)

/singleselect {

optional: false,

default_value: "-1",

dbcolumn: "SVS_1_Power",

inline:true,

colalign:false,

options: [[{dbvalue: "8", text: "8 (Of supreme importance)"},

{dbvalue: "7", text: "7"},

{dbvalue: "6", text: "6"},

{dbvalue: "5", text: "5"},

{dbvalue: "4", text: "4 (Important)"},

{dbvalue: "3", text: "3"},

{dbvalue: "2", text: "2"},

{dbvalue: "1", text: "1 (Not important)"},

{dbvalue: "0", text: "0 (Opposed to my principles)"},

]]

}

/p

/spacer

/lb

#6

6. UNIVERSALISM (broad-mindedness, beauty of nature and arts, social justice, a world at peace, equality, wisdom, unity with nature, environmental protection)

/singleselect {

optional: false,

default_value: "-1",

dbcolumn: "SVS_1_Power",

inline:true,

colalign:false,

options: [[{dbvalue: "8", text: "8 (Of supreme importance)"},

{dbvalue: "7", text: "7"},

{dbvalue: "6", text: "6"},

{dbvalue: "5", text: "5"},

{dbvalue: "4", text: "4 (Important)"},

{dbvalue: "3", text: "3"},

{dbvalue: "2", text: "2"},

{dbvalue: "1", text: "1 (Not important)"},

{dbvalue: "0", text: "0 (Opposed to my principles)"},

]]

}

/p

/spacer

/lb

#7

7. BENEVOLENCE (helpfulness, honesty, forgiveness, loyalty, responsibility)

/singleselect {

optional: false,

default_value: "-1",

dbcolumn: "SVS_1_Power",

inline:true,

colalign:false,

options: [[{dbvalue: "8", text: "8 (Of supreme importance)"},

{dbvalue: "7", text: "7"},

{dbvalue: "6", text: "6"},

{dbvalue: "5", text: "5"},

{dbvalue: "4", text: "4 (Important)"},

{dbvalue: "3", text: "3"},

{dbvalue: "2", text: "2"},

{dbvalue: "1", text: "1 (Not important)"},

{dbvalue: "0", text: "0 (Opposed to my principles)"},

]]

}

/p

/spacer

/lb

#8

8. TRADITION (respect for tradition, humbleness, accepting one's portion in life, devotion, modesty)

/singleselect {

optional: false,

default_value: "-1",

dbcolumn: "SVS_1_Power",

inline:true,

colalign:false,

options: [[{dbvalue: "8", text: "8 (Of supreme importance)"},

{dbvalue: "7", text: "7"},

{dbvalue: "6", text: "6"},

{dbvalue: "5", text: "5"},

{dbvalue: "4", text: "4 (Important)"},

{dbvalue: "3", text: "3"},

{dbvalue: "2", text: "2"},

{dbvalue: "1", text: "1 (Not important)"},

{dbvalue: "0", text: "0 (Opposed to my principles)"},

]]

}

/p

/spacer

/lb

#9

9. CONFORMITY (obedience, honoring parents and elders, self-discipline, politeness)

/singleselect {

optional: false,

default_value: "-1",

dbcolumn: "SVS_1_Power",

inline:true,

colalign:false,

options: [[{dbvalue: "8", text: "8 (Of supreme importance)"},

{dbvalue: "7", text: "7"},

{dbvalue: "6", text: "6"},

{dbvalue: "5", text: "5"},

{dbvalue: "4", text: "4 (Important)"},

{dbvalue: "3", text: "3"},

{dbvalue: "2", text: "2"},

{dbvalue: "1", text: "1 (Not important)"},

{dbvalue: "0", text: "0 (Opposed to my principles)"},

]]

}

/p

/spacer

/lb

#10

10. SECURITY (national security, family security, social order, cleanliness, reciprocation of favors)

/singleselect {

optional: false,

default_value: "-1",

dbcolumn: "SVS_1_Power",

inline:true,

colalign:false,

options: [[{dbvalue: "8", text: "8 (Of supreme importance)"},

{dbvalue: "7", text: "7"},

{dbvalue: "6", text: "6"},

{dbvalue: "5", text: "5"},

{dbvalue: "4", text: "4 (Important)"},

{dbvalue: "3", text: "3"},

{dbvalue: "2", text: "2"},

{dbvalue: "1", text: "1 (Not important)"},

{dbvalue: "0", text: "0 (Opposed to my principles)"},

]]

}

/p

/spacer

/lb

/p

When you have responded to <u>all</u> the questions, please click

/ts + bold

SUBMIT

/ts - bold

to proceed.

A warm thank you for taking part in our study!

/p

/p

This study included two groups, those with experience in meditation and those with experience in psychedelics. The aim of the study is to examine the relationship between peak experiences and wellbeing.

/p

/p

If you are interested in the results of the study, please keep an eye on the website <a href="https://www.abo.fi/prn">www.abo.fi/prn</a>. Please note that it can take up to two years before the results are published.

/p

/p

If you have any questions regarding this study, please contact us at jjylkka@abo.fi. All correspondence is handled confidentially.

/p

/p

Researchers at Turku University and Åbo Akademi University,

/p

Turku, Finland

/p

/p

/p

Please click on

/ts + bold

SUBMIT

/ts - bold

to finish the survey.

/p
